# Cognitive Flexibility Improves Memory for Delayed Intentions

**DOI:** 10.1523/ENEURO.0250-19.2019

**Published:** 2019-11-01

**Authors:** Seth R. Koslov, Arjun Mukerji, Katlyn R. Hedgpeth, Jarrod A. Lewis-Peacock

**Affiliations:** 1 Department of Psychology; 2 Institute for Neuroscience, University of Texas, Austin, Texas 78712; 3 Helen Wills Neuroscience Institute, University of California, Berkeley, Berkeley, California 94720

**Keywords:** cognitive control, cognitive flexibility, fMRI, MVPA, prospective memory, working memory

## Abstract

The ability to delay the execution of a goal until the appropriate time, prospective memory (PM), can be supported by the following two different cognitive control strategies: proactive control involving working memory maintenance of the goal and active monitoring of the environment; or reactive control relying on timely retrieval of goal information from episodic memory. Certain situations tend to favor each strategy, but the manner in which individuals adjust their strategy in response to changes in the environment is unknown. Across two experiments, human participants performed a delayed-recognition PM task embedded in an ongoing visual search task that fluctuated in difficulty. A control strategy was identified from moment to moment using reaction time costs and fMRI measures of goal maintenance. We found that people fluidly modified control strategies in accordance with changes in task demands (e.g., shifting toward proactive control when task difficulty decreased). This cognitive flexibility proved adaptive as it was associated with improved PM performance.

## Significance Statement

Adapting to changes in the environment is important for achieving immediate goals, and it is also essential for remembering to perform future intentions. Using brain imaging and behavioral measures of cognitive control, we discovered that people fluidly shift between proactive and reactive control strategies, from moment to moment, in accordance with changes in ongoing task demands to successfully fulfill future intentions. These flexible shifts in control strategy were associated with better memory for delayed intentions, demonstrating that fine-grained control of attention and memory resources serves an adaptive role for remembering to carry out future plans.

## Introduction

Life is busy, and keeping track of what we are doing and what we intend to do can be challenging. Cognitive control describes the set of processes by which we are able to maintain and connect goals to actions and to subsequently filter out irrelevant distractors in accordance with these goals (Gratton et al., 2018). Juggling goals despite interruptions, an ability known as prospective memory (PM), is a ubiquitous part of everyday life (Dismukes, 2012), constituting upwards of 50–80% of our daily memory problems (Crovitz and Daniel, 1984; Kliegel and Martin, 2003). The multiprocess theory of PM (McDaniel and Einstein, 2000; Einstein and McDaniel, 2005) describes the following two dissociable strategies that can be used: proactive control and reactive control (see also Braver, 2012). Proactive control relies on working memory to remember the goal and external attention to monitor the environment for cues to act (McDaniel and Einstein, 2000; Guynn, 2003; Smith, 2003; Brewer et al., 2010). Reactive control relies on episodic memory to store the goal and salient cues from the environment to trigger its timely retrieval (McDaniel and Einstein, 2007; Einstein and McDaniel, 2010; Marklund and Persson, 2012).

These strategies have been shown to have distinct behavioral and neural profiles. Proactive control is cognitively demanding (Braver et al., 2007; Cohen, 2008) and interferes with ongoing (OG) processing (Smith, 2003; Smith et al., 2007), whereas reactive control relies less on working memory processing and can succeed without any observable interference costs (Harrison and Einstein, 2010; Scullin et al., 2010a,b; Knight et al., 2011; Rummel and Meiser, 2013; Harrison et al., 2014). Dissociable neural correlates have also been identified for these strategies (Reynolds et al., 2009; Beck et al., 2014; Cona et al., 2014; McDaniel et al., 2013; Lewis-Peacock et al., 2016).

Preparatory processes involved in proactive control may benefit PM, but also place high costs on working memory and attentional capacities. In low-demand environments, controlled attentional processes can be successfully allocated to maintain goal information in working memory and to strategically monitor the environment for the right time and place to act (West et al., 2006, 2007b; Braver et al., 2007). However, in high-demand environments, it is more efficient to offload the PM intention to the reactive control system and redirect cognitive resources toward more immediate demands (Braver, 2012). While reactive control is less cognitively demanding, it is more susceptible to proactive interference and more vulnerable to lapses in attention to goal-relevant events in the environment (Braver et al., 2007; Scullin et al., 2012). It is therefore important to select a control strategy best suited to the current situation to reduce the risk that prospective intentions interfere with more urgent demands, and also to reduce the risk that those intentions go unfulfilled.

One major area of research in PM has been to explain how individuals choose strategies in response to variable environmental demands (Anderson et al., 2017). The dynamic multiprocess view (DMPV) (Scullin et al., 2013; Shelton and Scullin, 2017) proposes that people have a flexible choice between proactive control and reactive control that primarily depends on the contextual likelihood of a PM event. According to this model, individuals will rely on reactive control when the probability of a PM event is low, and then abruptly “switch on” proactive control when an environmental cue signals an increased likelihood of a PM event (Kuhlmann and Rummel, 2014; Ball et al., 2015; Cohen et al., 2017). However, previous work has primarily relied on blocked experiment designs or taken an all-or-none approach to measuring PM strategy. The environment may not always change abruptly, but instead may fluctuate more gradually from moment to moment. Correspondingly, PM strategy may fluctuate gradually between varying degrees of proactive and reactive control in response to the changing demands in the environment. The present study sought to test this hypothesis and to evaluate the impact of strategy flexibility on PM performance.

In this study, we tested the dynamics of PM strategy use by evaluating how individuals adjust their PM strategies when cognitive demands subtly increase or decrease over time, and in turn how this impacts prospective remembering. We hypothesized that adapting one’s control strategy to better align with the cognitive load caused by competing demands should improve prospective remembering. Participants made a delayed target detection with pictures of faces and scenes (the PM task) while also performing an ongoing visual search task with oriented arrows. The cognitive demands of the ongoing task were manipulated by subtly, but monotonically, adjusting task difficulty every couple of seconds. We linked time-sensitive behavioral measures of PM strategy shifts and multivariate neural measures of PM intention processing to memory performance on a trial-by-trial basis.

## Materials and Methods

Human subjects were recruited from the student body at the University of Texas at Austin as well as from the surrounding community, and the experiments were conducted in a manner consistent with the approval of the Internal Review Board of our institution. For Experiment 1, 55 participants were recruited. Five of these participants were excluded due to below chance (<50%) performance on the ongoing task across the experiment. For Experiment 2, we recruited 30 participants, 2 of whom were excluded for excessive movement during scanning that led to MR images failing quality control. Data analyses were performed on the remaining 78 participants (neural sample: 28 participants; 17 females; mean age, 21.8 years; behavioral sample: 50 participants; 31 females; mean age, 19.2 years).

**Figure 1.**
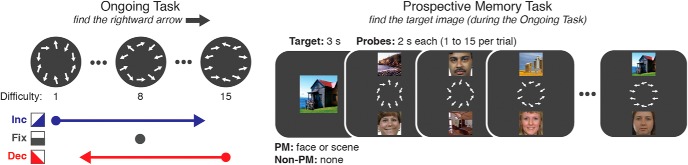
Task design. Left, Ongoing task difficulty could increase or decrease every 2 s across a trial or remain fixed at the middle difficulty level (level 8 of 15). We validated the relationship between difficulty levels in a pilot study, finding that as difficulty increased, reaction time increased and accuracy decreased. For more information on pilot study 1, see Extended Data Figure 1-1. We further validated that the ongoing task could impact prospective memory strategy use in a second pilot study, where we found PM cost was significantly higher at an easy difficulty (level 4) than at a harder difficulty (level 12). For more information on pilot study 2, see Extended Data Figure 1-2. Right, In the dual-task PM experiment, participants identified the reappearance of a PM target while concurrently performing the ongoing task.

Participants performed an ongoing visual search task with an embedded PM task (Fig. 1). For the behavioral sample, participants performed six blocks of the PM task. For the neural sample, participants first completed one PM practice block outside of the scanner to familiarize themselves with the dual-task paradigm. During the practice block, face and scene stimuli were replaced with tools and vehicles so that participants did not familiarize themselves with the main task stimuli, but all other task configurations remained the same. Following completion of the practice session, participants were asked to explain the task, and the researcher answered any lingering questions before placing the subject in the MR scanner.

10.1523/ENEURO.0250-19.2019.f1-1 Figure 1-1 Average reaction time (y-axis left, blue) and accuracy (y-axis right, red) for pilot participants are plotted across each difficulty level of the ongoing visual search task. Reaction time increases and accuracy decreases from the easiest difficulty (1) to the hardest (14). The purpose of the first behavioral pilot study was to determine if the ongoing task could be parametrically modulated in a controlled manner. For this pilot study, participants (n = 15) performed the ongoing task in isolation. On each probe, participants indicated the absence or presence of the arrow target (rightward facing horizontal arrow) in a newly generated visual-search array (every 2 s) with a button press (left: absent; right: present; response deadline: 1.9 s). Target arrow location was counterbalanced between the top and bottom half of the screen. Non-target arrows appeared in set positions around the circular array, oriented within some distribution of angles determined by the current task difficulty setting. Participants sat approximately 18 in. away from the screen, and all 10 arrows, which were .64° by .22° in shape, were 3.18° away from the center of the screen. OG task difficulty was manipulated on each probe by adjusting two parameters controlling the orientation of the distractor arrows: their minimum similarity to the target and their similarity to other distractors. For distractor-to-target similarity, a minimum angular distance for distractors from the target (i.e., horizontal or 0°) was set to either 5, 15, 25, 45, or 65 degrees. For distractor-to-distractor similarity, the maximum variance from the minimum angular distance was set to either 10, 20, or 40 degrees. The factorial combination of these parameters (excluding any combination where minimum plus variance could exceed the 90° vertical plane) created 14 difficulty conditions. Participants performed three blocks of trials separated by short voluntary breaks for rest. Each block contained 14 mini-blocks comprised of 20 visual-search trials of one difficulty level. Difficulty level was pseudo-randomly selected, with the only limitation being that each of the 14 difficulty levels was selected exactly three times. As difficulty increased, accuracy decreased (F(13,182)=74.89, p<.001) and reaction time increased (F(13,182)=39.53, p<.001). Download Figure 1-1, DOCX file.

**Figure 2.**
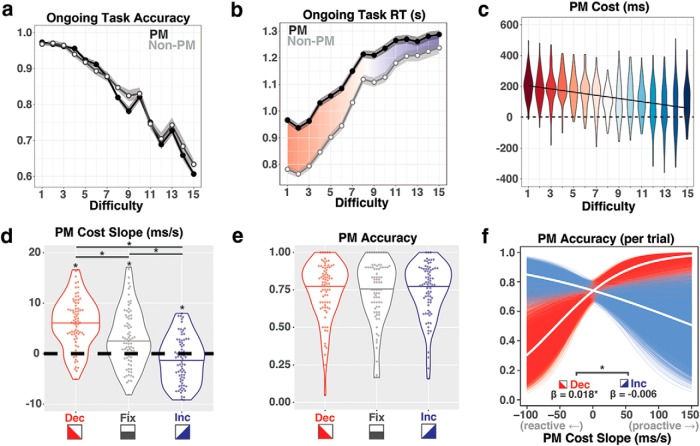
Behavioral performance. ***a***, Ongoing task accuracy across difficulties, error ribbons ± 1 SEM. PM, Dual-task trials with a PM intention; Non-PM, ongoing task only without a PM intention. ***b***, Ongoing task RT (correct responses only) across difficulties, error ribbons ± 1 SEM. ***c***, PM cost (the difference between ongoing task RT for PM trials vs Non-PM trials) was computed for each participant at every difficulty level. Violin plots represent the distribution of by-participant average costs at each difficulty. PM cost is highest at easy difficulty levels (dark red) and decreases as task difficulty increases (dark blue). ***d***, Polynomial model fits validated the use of linear models, which allowed us to calculate the shift in PM cost on each trial (for further analysis details, see text below, Extended Data Figure 2-1, Extended Data Table 2-3). Next, PM cost slopes were calculated as the change in PM cost within each trial. Violin plots show the average within-trial PM cost slopes for decreasing (Dec), fixed (Fix), and increasing (Inc) trials across participants. *p < 0.05. ***e***, PM accuracy for each participant across trial types. ***f***, Logistic regression bootstrap analysis linking PM cost slope to PM accuracy for decreasing (red) and increasing (blue) trials. Each individual red/blue line shows the predicted relationship for each bootstrapped sample (*n* = 10,000). White lines reflect the fixed-effects relationship for the original sample. *p < 0.05.

10.1523/ENEURO.0250-19.2019.f1-2 Figure 1-2 The purpose of the second behavioral pilot study was to determine if PM strategy (as measured by PM cost) could be modulated by the difficulty of the ongoing task. The task design for this study was nearly identical to that used in the main experiment, but here OG task difficulty was held constant as either *easy* (difficulty level 4) or *hard* (difficulty level 12) for the entirety of each block and across each trial. Participants (n = 20) completed one block (15 trials per block) at each difficulty level. **a)** PM cost was calculated by subtracting the average non-PM trial OG RT from the average PM trial OG RT at each difficulty level for each participant. The distribution and median are represented. PM cost significantly varied as a function of OG task difficulty (F(1,19) = 35.63, p<.001), with cost being higher at the easier difficulty (M = 0.134 s (SE = 0.012)) than at the harder difficulty (M = 0.031 s (SE = 0.012)). * p<.05. **b)** PM accuracy was calculated at each difficulty for each participant. The distribution and median are represented. PM accuracy was equivalent across conditions (F(1,19) = 0.785, p = 0.387; easy = 71.0% (4.5%), hard = 64.5% (5.8%)). Download Figure 1-2, DOCX file.

### Ongoing task details

The ongoing task was a visual search task (“OG task”) where participants searched for a specific target arrow on a circular array of oriented arrows (Fig. 1*A*). We chose this as the OG task due to the ability to systematically and parametrically manipulate task difficulty by adjusting distractor parameters along a continuum (Sobel et al., 2007; Kiyonaga et al., 2017). The ongoing task target arrow was always a rightward facing horizontal arrow (→). The target arrow was present on a randomly selected half of the trials, located in one of 10 semirandomly selected locations around the circle. Participants were instructed to search for the target on each display and use their right hand to press “1” for present and “2” for absent (for the MRI sample, that was buttons 1 and 2 on the response box). Target arrow location was counterbalanced between the top and bottom halves of the screen. On “present” trials, 9 nontarget (distractor) arrows appeared in set positions around the circular array (10 on “absent” trials), oriented within some distribution of angles determined by the current task difficulty setting.

OG task difficulty was manipulated on each probe by adjusting two parameters that determined the orientation of the distractor arrows, as follows: their minimum similarity to the target, and their similarity to other distractors. For distractor-to-target similarity, a minimum angular distance was set to either 5°, 15°, 25°, 45°, 65°, or 75°. For distractor-to-distractor similarity, the maximum variance from the minimum angular distance was set to 10°, 20°, or 40°. The factorial combination of these parameters (excluding any combination where minimum distance plus variance could exceed the 90° vertical plane) created 15 difficulty conditions. On every search display, each distractor arrow had a 50% chance of being flipped across the horizontal plane and a 50% chance of being flipped across the vertical plane, so that distractor arrows could vary from 5–175° or 185–355°. To minimize uncontrolled pop-out effects, we ensured that no distractor arrow in the array was within 5° of an arrow that appeared in the same location during the previous display. We validated the relationship between difficulty levels in a pilot study, finding that as difficulty increased, reaction time increased (*F*_(13,182)_ = 39.53, *p* < 0.001) and accuracy decreased (*F*_(13,182)_ = 74.89, *p* < 0.001). For more information on the pilot study, see Extended Data Figure 1-1.

For the behavioral sample, participants sat ∼18 inches away from the screen, and all 10 arrows, which were 0.64° by 0.22° in shape, were 3.18° away from the center of the screen. For the MRI sample, stimuli were projected on to a screen which participants viewed through a mirror placed over the headcoil. Participants laid face up, looking at a projection screen that was ∼136 cm away from the mirror. To keep the stimulus proportions relative to the field of view the same across experiments, projected arrows were 30° × 11°, and placed 1.39° away from the center of the screen.

### PM task stimuli

Colored images of unfamiliar faces and unfamiliar scenes were gathered from various in-house and on-line sources. These images were controlled for valence and familiarity. Of those images, 230 (115 faces, 115 scenes) were selected for use in this experiment. For each participant, 40 faces and 40 scenes were randomly selected to serve as the PM targets and, 75 faces and 75 scenes were used as distractors. PM target images did not appear as distractors and were used on one trial only. Distractor images never reappeared within the same trial, but later reappeared on subsequent trials (mean exposures per distractor, 14; minimum, 6; maximum, 20).

### PM task description

Each trial began with the presentation of the PM target (a face, a scene, or no PM target) for 3 s, followed by a 1 s fixation cross. For non-PM trials, participants saw a yellow null (∅) sign in lieu of a face or scene. For PM trials, participants were informed that the PM target shown was only relevant for the current trial. After target presentation, participants saw a series of 1–15 probes per trial. Every probe contained a visual search array in the center of the screen, with one face and one scene (each one 9.5° × 9.5° in visual angle) vertically aligned with the center of the images placed 11.5° above or below the search array. Each probe was on the screen for 2 s, during which the participants were allowed 1.9 s to respond to the presence or absence of the horizontal arrow in the OG task or to indicate whether the PM target had reappeared (by pressing “3” for behavioral participants or the third button on the button box for MRI participants). Participants were instructed to equally weight the importance of both tasks, and only one response (to either the OG task or the PM task) was allowed per probe (“task-switch” approach; Bisiacchi et al., 2009). Visual feedback was presented immediately following each response in the form of the arrows turning green for correct OG responses, turning red for incorrect OG responses, or a yellow border surrounding the screen for PM false alarms. Probe feedback remained on screen for the remaining duration of each 2 s probe. The 1.9 s response deadline ensured that some time (minimum, 100 ms) was always devoted to feedback on every probe. On trials with a PM target presented at the beginning of the trial (“PM trials”), participants performed both tasks, as described above. On trials with no PM target presented (“non-PM trials”), participants were instructed to ignore the face and scene images and focus solely on the OG task. The PM target reappeared only once per trial, and its reappearance always marked the end of the trial. After the final probe of each trial, participants were given feedback on the PM task in the form of a green border appearing around the edge of the screen for correct PM responses and a red border for missed PM targets. This feedback (or a blank screen for non-PM trials) remained for 2 s and was followed by a 6 s rest interval with a fixation cross on the screen before the next trial began.

PM and non-PM trials were randomly intermixed within each block, with one-third of all trials being non-PM trials, and the other two-thirds were equally split between face–target or scene–target PM trials. Participants were able to rest between blocks for as long as they wanted before continuing with the experiment. OG task difficulty was manipulated in the following five conditions: it could (1) increase starting at the easiest difficulty (level 1), (2) increase starting at the median difficulty (level 8), (3) decrease starting at the hardest difficulty (level 15), (4) decrease starting at the median difficulty, or (5) remain fixed at the median difficulty. For the main analyses reported here, the first two conditions were combined as “increasing” trials, the second two conditions were combined as “decreasing” trials, and the fifth condition was referred to as “fixed.” Each of the five difficulty types occurred three times throughout each block in pseudorandom order. Fifteen trials corresponding to the 3 × 5 combinations of PM type (face/scene/non-PM) and OG difficulty type occurred once per block. Five trials in each block were “catch trials” (containing fewer than eight probes), which were included to keep participants engaged at the beginning of each trial. The difficulty types and target category for catch trials were counterbalanced across the entire experiment. Trial lengths were predetermined and pseudorandomized so that every participant had the same number of total probes for face–target, scene–target, and non-PM trials. Face and scene locations were randomized on each probe, and faces and scenes appeared on the top or bottom of the display with equal probability. Performance on face and scene trials was similar, so to increase statistical power, these trials were collapsed for subsequent analyses.

Changes in difficulty occurred at the rate of one shift in difficulty level per probe until either the end of the trial or until a difficulty end point was reached. For example, on a 10-probe trial starting at the easiest difficulty (level 1) and then increasing in difficulty, the trial would end at difficulty level 10. However, on a 10-probe trial starting at the median difficulty (level 8) and then increasing in difficulty, the highest difficulty (level 15) would be reached by the eighth probe, and the difficulty would remain at this level for the final two probes of the trial. For the behavioral experiment, this led to 70 PM probes at the hardest (level 15) and easiest (level 1) difficulty levels, 210 PM probes at the middle difficulty (level 8), and 30–40 PM probes at the other difficulty levels across the entire experiment. There were approximately half that many non-PM probes at each level. For the MRI sample, in which there was one less experimental block, participants had an average of 53 probes at the hardest and easiest difficulties, 169 at the middle difficulty, and 22–34 at the other difficulties. For non-PM probes, the count across the experiment was approximately half of those totals.

### Ongoing task localizer (MRI sample only)

Once in the scanner, each participant first completed the OG arrow visual search task. The OG task localizer provided participants with the chance to familiarize themselves with this task in the MRI setting before the addition of the embedded PM task. It also allowed for us to collect time points where only the OG task was displayed on the screen for training the classifiers on non-PM probes. The arrow search task was the same as during the PM task; however, there were no face or scene images on the screen during the OG task localizer. Participants indicated the presence or absence of a target right-facing horizontal arrow within a 1.9 s response window. Feedback was given in the form of arrows turning green for correct responses and red for incorrect responses through the end of the response window. Similar to the main task, the difficulty of the OG task gradually increased or decreased over the course of a trial. Each block included 60 probes, split into eight trials of various lengths (minimum = 2, maximum = 12). There was a 6 s rest interval between trials. Participants performed two blocks of the ongoing task localizer (120 probes total) before moving on to the main PM task.

### Face/scene localizer (MRI sample only)

After completion of the main PM task and a quick subsequent memory test (not discussed here), participants performed a face/scene subcategorization localizer task. This localizer allowed for us to collect more data points of face and scene processing for training the fMRI pattern classifiers. Presentation of faces and scenes alternated in mini-blocks, where 11 stimuli from the same category (faces or scenes) were presented in a row, and participants indicated whether a face was male or female or whether a scene was indoors or outdoors. A single face or scene image was presented in the center of the screen, and participants had 1s to respond. Because of the short response window, female/outdoor was always presented as the left option, and male/indoor was always presented as the right option. Note that this corresponded to button box responses “1” and “2” in the scanner, which did not overlap with the PM-repsonse button “3”. Immediately following a response, a red (incorrect) or green (correct) box appeared around the selected choice, or a blue box (no response) appeared around the correct choice for 500 ms. The stimulus remained on the screen during this time. There were then 500 ms between trials during which a fixation cross appeared on the screen. There were three blocks with six mini-blocks pseudorandomly interleaved for a total of 198 localizer trials. While the fMRI pattern classifiers performed well without the inclusion of the data from both localizers, they performed numerically better when localizer data were included. Therefore, our final analysis included samples from both the OG localizer task as well as the face/scene localizer task.

### MRI acquisition and preprocessing

MRI data were acquired on a 3.0 T Siemens Skyra MRI scanner with a 32-channel head coil. Whole-brain, high-resolution anatomic images were collected for registration and parcellation using a T1-weighted MPRAGE sequence [repetition time (TR) = 1900 ms; echo time (TE) = 2.43 ms; flip angle = 9°; field of view (FOV) = 256 × 256 × 192; 1 mm isotropic voxels]. Functional images were acquired using a T2*-weighted multiband accelerated EPI pulse sequence (TR = 2 s; TE = 29 ms; flip angle = 78°; FOV = 76 × 76; slice thickness = 3 mm; multiband factor = 2; number of slices = 48; no gap; 3 mm isotropic voxels). Following shim adjustment at the beginning of the scan session, a B0 field map with the same slice prescription as the functional data was acquired.

As an initial step for preprocessing, DICOMs (Digital Imaging and Communications in Medicine) were converted to NIFTI (Neuroimaging Informatics Technology Initiative) format using dcm2niix (Li et al., 2016). Next, the recon-all function in Freesurfer (Fischl, 2012) was used to skull strip and parcellate the high-resolution anatomic image. MRI data were preprocessed using a combination of functions in Freesurfer 6.0, FSL 5.0.9 (Jenkinson et al., 2012), and ANTs 2.1.0 (Avants et al., 2011). Functional images were first slice time corrected using the FSL slicetimer function. Then, functional runs were normalized to the third run of the main task (middle run) using a combination of within-run motion correction, rigid and affine registration, and field unwarping processes from FSL and ANTs. Nonlinear registration, via antsRegistrationSyn, was then used to correct for between run differences. Last, high-pass filtering (128 s) was applied to the images.

**Figure 3.**
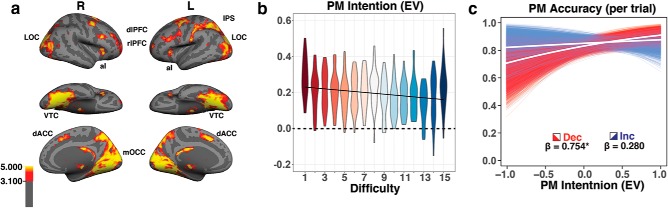
fMRI decoding of PM intentions. ***a***, Brain regions significantly engaged by the addition of the PM task to the OG task (GLM contrast PM > Non-PM, FDR corrected at *p* < 0.001; Extended Data Table 3-1, ROI list). aI, Anterior insular cortex, dACC, dorsal anterior cingulate cortex, dlPFC, dorsolateral prefrontal cortex, IPS, Intraparietal sulcus, LOC, lateral occipital cortex, mOCC, medial occipital cortex, rlPFC, rostrolateral prefrontal cortex, VTC, ventral temporal cortex. These regions were used as the initial feature mask to train and test fMRI pattern classifiers for PM intention-related activity. To more directly identify regions primarily responsible for PM intention representation during this task, we performed a surface-based searchlight analysis. That analysis indicated that the VTC and LOC were more important for PM processing (for more details, see Extended Data Figure 3-2). ***b***, PM EV (the difference between classifier evidence for the category of the PM target and the nontarget category) was computed for each participant at every difficulty level, and group data are shown in violin plots. PM intention evidence was highest at easy difficulties (dark red) and lowest for the most difficult levels (dark blue). ***c***, The relationship between PM intention evidence and PM accuracy was computed using bootstrapped logistic regression (*n* = 10,000 iterations) for decreasing (red) and increasing (blue) trials. *p < 0.05.

For the univariate GLM (General Linear Model) analysis, rigid and affine transformations were used to register the functional scans to the high-resolution anatomic, and then nonlinear transformations were applied to normalize runs to the MNI template. These images were spatially smoothed (5 mm Gaussian), but no further preprocessing was performed before using FSL FEAT for modeling. The model included separate regressors for the PM target presentation, PM trial probes 1 to *n* − 1, probe *n* (when the PM target reappeared in PM trials), non-PM trial probes 1 to *n*, trial feedback, and rest. Six motion parameters, extracted using MCFLIRT in FSL, were included as confound regressors. FEAT in FSL was used to identify voxels that were more responsive on PM probes than on non-PM Probes (cluster correction, *p* < 0.001; Fig. 3*A*, Extended Data Table 3-1, ROI list).

This group level results map was then individually transformed from standard space into subject functional space so that the multivariate pattern analysis could be performed independently for each participant. Individual, native-space masks were created by applying a reversed transformation matrix from EPI to MNI stereotaxic space on the group-level GLM mask described above.

The classification analysis was performed using the Princeton MVPA toolbox in MATLAB (https://github.com/princetonuniversity/princeton-mvpa-toolbox). Binary (one vs all other classes) L2-penalized logistic regression classifiers (penalty = 50) were trained, separately for each participant, to differentiate fMRI activity corresponding to faces, scenes, the OG task, and resting periods in between trials. A combination of data from the OG task localizer (labeled as class “OG”), from the face/scene localizer (labeled as class “face” or “scene”), and from the PM task (labeled as “face,” “scene,” or “OG,” depending on the PM target for each trial) were used to train the classifiers using k-fold cross-validation. From the main PM task, data from all probes (except for the final probe on each trial when the PM target reappeared) were used for training. For the k-fold cross-validation procedure, classifiers were trained on all data from the two localizer tasks plus four of the five runs from the PM task. In total, there were 7180 time points used for classifier training on each iteration (face = 1695, scene = 1695, OG = 1900, rest = 1890), and 1215 used for testing on each iteration (face = 300, scene = 300, OG = 325, rest = 290). All regressors were shifted forward in time by two TRs (4 s) to account for the hemodynamic lag. These classifiers were then applied to the held-out run of data from the PM task. The PM task runs were then rotated, and this procedure was repeated to train classifiers and then test them on the next held-out task run. This procedure was performed five times so that all runs of the PM task were tested. To improve classifier accuracy, we performed feature selection to remove uninformative voxels from the training data. This was done separately for each fold of the cross-validation analysis. Data within each voxel were *z*-scored across all time points, and a 1 × 4 ANOVA was performed to select only those voxels that demonstrated significant (*p* < 0.05) variance across the four classes being trained (face, scene, OG, and rest). The mean number of voxels selected for each participant was 7864 voxels (SEM = 1208).

Each of the 1-versus-other classifiers produced an evidence score for the class on which it was trained. Therefore, the four evidence values produced for each test time point need not sum to one. At each time point, the class with the highest evidence value was selected as the predicted output. These predicted outputs were compared with the actual class of the time point (face, scene, OG, or rest) to calculate the classifier accuracy. The area under the curve (AUC) was calculated in MATLAB by comparing correct predictions and false alarms independently for each category across all time points. Scrambled regressor assignments were used to test the empirical chance level of classifier performance trained in this way (*n* = 1000/participant). Average scrambled baseline performance (mean = 27.52%) was similar to the empirical chance level of 25% for all participants.

### Calculating PM cost and PM cost slope

For all analyses involving response times (RTs), we excluded any responses faster than 300 ms. This criterion is in line with previous work (Boywitt and Rummel, 2012; Horn et al., 2013) and was used to exclude late responses carried over from the preceding response window. We hypothesized that individuals would be able to adjust their PM strategy on a moment-to-moment basis in response to fluctuating cognitive demands. To initially test this theory, we performed a second pilot study where we had participants perform our PM task at either an easy (level 4 in the main study) or hard (level 12 in the main study) OG difficulty level. We found that PM cost significantly varied as a function of OG task difficulty (*F*_(1,19)_ = 35.63, *p* < 0.001), with cost being higher at the easier difficulty (easy PM cost: mean = 0.134 s; SE = 0.012) than at the harder difficulty (hard PM cost: mean = 0.031 s; SE = 0.012). PM accuracy was equivalent across difficulties [*F*_(1,19)_ = 0.785, *p* = 0.387; easy = 71.0% (4.5%); hard = 64.5% (5.8%)]. For more information about pilot study 2, see Extended Data Figure 1-2. In the current study, we calculated the PM cost at each task difficulty level associated with making a correct response on the OG task with versus without the additional demand of the PM task (i.e., PM trials vs non-PM trials).

To calculate a PM cost for each probe, we first calculated the average OG RT on non-PM probes at each level of difficulty. We performed this analysis separately for each participant. We included only correct OG responses; however, a control analysis including all OG task responses produced qualitatively similar results. There was a practice effect of decreasing overall RTs between early experimental blocks and late experimental blocks (*F*_(1,77)_ = 87.1, *p* < 0.001). To account for these practice effects, we calculated his baseline separately for the first half (early) and second half (late) of the experiment. For the behavioral sample in Experiment 1, early trials came from blocks 1–3, and late trials from blocks 4–6. The MRI sample in Experiment 2 had one practice block outside of the scanner (data not recorded), so for those participants early trials came from main task blocks 1–2, and late trials from blocks 3–5. This protocol created 30 baseline values for each participant, as follows: 15 difficulties × 2 time bins (early/late). To create the PM cost for each individual probe from PM trials, we first determined the relevant baseline value (the value with the same difficulty and time bin as the probe) and subtracted that value from the OG RT on that probe. This enabled us to estimate the PM cost for every probe of the experiment. After obtaining PM cost values for each probe, we then calculated the degree to which PM cost shifted over the course of each trial (PM cost slope). The PM cost slope was determined by calculating the difference in average PM costs of the first three probes versus the final three probes of a trial (excluding the very last probe in which the PM target appeared), and then dividing by the number of probes in the trial. To account for lapses in motivation and to exclude trials where there was a lack of correct OG probes for calculating a PM cost slope, we excluded any trial where OG task accuracy fell below the chance level of 50% [this led to an average of 9.9% (SE = 1.0%) of trials per participant being excluded].

Because this paradigm involves long PM trials (mean, 28 s; range, 8–36 s), it produced few behavioral PM reports per participant. Therefore, to increase statistical power, we performed a nonparametric bootstrap analysis (Efron, 1979) using data sampled from all participants (for for examples see: Kim et al., 2010; Lewis-Peacock et al., 2016). On each bootstrap iteration (*n* = 10,000) of this analysis, 78 participants were selected at random with replacement. By using random selection with replacement, this form of analysis allows us to see to what extent the results are generalizable (Thompson, 1993). We used logistic regression to test the relationship between PM accuracy and PM cost slope and/or PM intention evidence (EV) on the different trial types. The stability of the effects across all iterations was analyzed to assess population-level reliability.

### Code accessibility

The code described in the article will be made freely available on-line at [https://osf.io/bqsc4/]. All code for this experiment was run using Psychophysics Toolbox version 3 in Matlab 2014b on a 21.5 inch iMac computer with operating system OSX 10.11. All statistical analyses of behavioral data were performed using R (version 3.4.1; R Core Team, 2017).

## Results

### Ongoing task performance

Participants performed well on the OG task (mean accuracy = 83.64%, 95% CI = 75.49–91.79%) as summarized in Figure 2 (Extended Data Table 2-3, compare experiments 1, 2). There was no interaction between trial type (PM trials vs non-PM trials) and task difficulty (β_interaction_ = 6.2 * 10^−4^, 95% CI = −0.001 to 0.002, *p*_interaction_ = 0.468) on OG task accuracy. OG accuracy (OG Acc) decreased as difficulty increased, and there was a small, but reliable, main effect of trial type between PM and non-PM trials (β_diff_ = −0.026, 95% CI = −0.027 to −0.025, *p*_diff_ < 0.001; β_pm_ = 0.008, 95% CI = 0.001–0.016, *p*_pm_ = 0.024; marginal *r* ^2^ = 0.57; Fig. 2*A*). The main effect of trial type indicated a dip in OG accuracy of <1% on PM trials compared with non-PM trials. A follow-up analysis compared OG accuracies from PM and non-PM trials at each difficulty level, finding that accuracies were only reliably different at difficulty level 8 on fixed-difficulty trials (*p* = 0.025, after Bonferroni correction factor 15). On dynamic trials (increasing and decreasing difficulty), the main effect of PM task on OG accuracy was not significant (β_pm_ = 0.007, 95% CI = −8.5 * 10^−5^ to 0.015, *p*_pm_ = 0.052). We also found that at the hardest difficulty level of the OG task (level 15), participants were still performing well above chance (mean = 61.43, *t*_(77)_ = 80.39, *p* < 0.001, 95% CI = 59.90–62.95%).

10.1523/ENEURO.0250-19.2019.f2-1 Figure 2-1 Visualization of model comparisons for by-trial estimates of PM strategy shifts. Here, we used AICc to evaluate the relative model fit between a linear, quadratic, and cubic relationship of PM cost over the course of each trial. The Akaike weight calculated from AICc scores from each model are shown in **Figure 2-3a**. We evaluated the lowest AICc for each trial, and then calculated the proportion of trials best fit by either a linear, quadratic, and cubic relationship for each participant (shown in **Figure 2-3b**). A 1st order polynomial (linear model) fit best for nearly all trials (mean = 93.43%, 95% CI = [92.62%, 94.24%]; Akaike weight = 0.873, 95% CI = [0.865, 0.880]), compared to 2nd order fits (mean = 5.72%, 95% CI = [4.99%, 6.45%]; Akaike weight = 0.111, 95% CI = [0.104, 0.119]), or 3rd order fits (mean = 0.85%, 95% CI = [0.68%, 1.02%]; Akaike weight = 0.016, 95% CI = [0.012, 0.019]). Download Figure 2-1, DOCX file.

10.1523/ENEURO.0250-19.2019.f2-2 Figure 2-2 Additional model comparisons for trial-by-trial estimates of PM strategy shifts. One concern with fitting models on a by-trial basis is that noise may bias model selection towards the models with fewer parameters. In order to address this concern, we performed a less conservative AIC (without the small sample correction term) model selection analysis and a bootstrap analysis where polynomial fits were calculated for a random subsample of trials. For the AIC analysis, we performed the same regression analysis steps as detailed for the AICc analysis, but simply used the AIC estimation term instead of the AICc term. After calculating an AIC score for each trial, we then selected the lowest score between the 1^st^, 2^nd^, and 3^rd^ order polynomial as the best for the trial. We then calculated the relative Akaike weight for each model on each trial and average that value for each model type for each participant. Across participants the average Akaike weights were similar between 1^st^ and 3^rd^ order polynomial fits (mean difference = 0.006 (SE = .010), t(77) = 0.65, p=.52; **panel a**). Significantly more trials for each participant were best fit by a 1^st^ order than a 3^rd^ order polynomial (mean difference = 20.5% (SE = 1.7%), t(77) = 12.014, p<.001; **panel b**). Another way to mitigate the impact of noise on model selection is to fit the model on more than a single trial at a time. To avoid averaging across all trials and still getting an estimate of model-fit reliability, we performed a bootstrap analysis. In this analysis, we first z-scored PM-cost values within each subject. Next, we combined all trials into one super-subject. On each bootstrap iteration, 50 trials of each of the five trial types (increasing starting easy, increasing starting middle, fixed, decreasing starting hard, decreasing starting easy) were randomly selected from the super-subject pool. Then, 1^st^, 2^nd^, and 3^rd^ order polynomial models were fit to each trial type sample and the lowest AIC value was selected as the best-fit model type. We repeated this process 1000 times and found that a linear (1^st^ order) polynomial fit a significantly greater number of these samples (57.7% of all trials), followed by a quadratic fit (2^nd^ order, 29.1% of all trials), followed by a cubic fit (3^rd^ order, 13.4% of all trials). This is a significantly greater number of trials selected to be best fit by a linear relationship than would be predicted by chance (χ^2^ (1, n=1000) = 364.06, p <.001). Download Figure 2-2, DOCX file.

Table 2-3 Comparison of behavioral results from experiments 1 and 2. These data include the key results presented in Figure 2 separately for the behavioral-only participants, the neural participants, and the combined groups. Each analysis section of the table (A-F) corresponds to the same panel from Figure 2. Analyses of the relationship between trial type (PM/non-PM), Difficulty (1 to 15) and OG task accuracy, OG RT, and PM cost were carried out by first running a mixed-effect regression using the lme4 package in R of the interaction between trial type and difficulty and then a separate model comparing the main effects of difficulty and trial type without the interaction term. Random effects of individual slope and intercept were included in each regression. Within-subject ANOVAs were used to compare PM cost slope and PM accuracy across conditions. Download Table 2-3, DOCX file.

One of the primary methods for inferring PM strategy use is measuring the difference in RTs between PM and non-PM trials on an ongoing task (Einstein and McDaniel, 2005). A large difference in OG RTs between PM and non-PM trials implies the use of proactive control, whereas a small difference implies the use of reactive control. Here, we found an interaction in OG RTs between condition (PM and non-PM trials) and difficulty level (levels 1–15; β_interaction_ = 0.011, *p* < 0.001, 95% CI = 0.010–0.013; Fig. 2*B*). At the hardest difficulty level, RTs were well below the response deadline of 1900 ms (*t*_(77)_ = 38.401, *p* < 0.001, mean RT = 1264 ms, 95% CI = 977–1551 ms), demonstrating that participants were performing below ceiling. The difference in OG RT between PM and non-PM trials (referred to as “PM cost” from here on) was then calculated for each participant at each difficulty level. These data were replotted in this fashion, and they reveal that average PM costs varied systematically as a function of OG task difficulty (β_cost_ = −10.35, *p* < 0.001, 95% CI = −12.29 to −8.42; Fig. 2*C*). This suggests that PM strategy shifted flexibly between proactive control and reactive control as the OG task increased in difficulty, and vice versa.

To evaluate whether this linear shift in PM strategy across task difficulty levels held within individual trials, we computed first-, second-, and third-order polynomial regressions between PM cost and OG difficulty separately for each trial. If PM strategy selection was bimodal (i.e., an all-or-none “flip” between proactive and reactive control), then individual trials should be best fit by a third-order polynomial. However, if the relationship was more fluid, then a first- or second-order polynomial should fit the data better. Additionally, if the data demonstrate a dramatic U-shaped or asymptotic curve, instead of a linear fit, a second-order polynomial should fit better than a first-order polynomial. We compared values for Akaike information criterion with a correction for small sample sizes (AIC_c_) for each model for correct responses on each trial and used Akaike weighting to compare relative model fits. We found that a first-order polynomial (linear model) fit best for nearly all trials [Extended Data Fig. 2-1; data visualization: mean = 93.43%, 95% CI = 92.62–94.24%; Akaike weight (wAIC) = 0.873, 95% CI = 0.865–0.880], compared with second-order fits (mean = 5.72%, 95% CI = 4.99–6.45%; Akaike weight = 0.111, 95% CI = 0.104–0.119), or third-order fits (mean = 0.85%, 95% CI = 0.68–1.02%; Akaike weight = 0.016, 95% CI = 0.012–0.019). One concern with fitting models on a by-trial basis is that noise may bias model selection toward the models with fewer parameters. To address this concern, we performed a less conservative AIC (without the small sample correction term) model selection analysis and then a separate bootstrap analysis where polynomial fits were calculated for a random subsample of trials. Both of these analyses corroborate our original finding and indicate that a linear fit is the most likely descriptor for a majority of trials (Extended Data Fig. 2-2). This result provides evidence that the engagement of different control strategies often changes fluidly and linearly in accordance with shifts in OG task demands within a PM trial.

Next, we evaluated whether changes in PM cost within a trial were different for increasing-, decreasing-, and fixed-difficulty trials. To do this, we computed a within-trial measure of linear shift in PM cost from the beginning of the trial to the end of the trial, which we shall refer to as “PM cost slope.” We found that PM cost slopes varied systematically across trial types (*F*_(2,154)_ = 47.02, *p* < 0.001; Fig. 2*D*). PM cost slopes were negative on increasing trials (mean = −1.14 ms/s, 95% CI = –2.24 to –0.42, *t*_(77)_ = 2.07, *p* = 0.042), and were positive on decreasing trials (mean = 6.03 ms/s, 95% CI = 5.44–6.62, *t*_(77)_ = 10.23, *p* < 0.001) and fixed trials (mean = 3.34 ms/s, 95% CI = 2.07–4.61, *t*_(77)_ = 5.24, *p* < 0.001). In this task design, a PM target appeared at the end of every PM trial. Thus, positive PM cost slopes (i.e., a shift toward proactive control) on fixed-difficulty trials likely arose from an increase in PM expectancy as each trial progressed (Oksanen et al., 2014; Bowden et al., 2017). Although expectancy should impact all trial types equally, there were meaningful differences between conditions. Planned pairwise comparisons revealed that PM cost slopes increased stepwise from increasing trials to fixed trials (*F*_(77)_ = 37.14, *p* < 0.001) and from fixed trials to decreasing trials (*F*_(77)_ = 15.1, *p* < 0.001).

### Prospective memory task performance

On average, participants identified the PM target on three-quarters of the trials (mean PM accuracy = 74.57%, 95% CI = 56.82–92.31%), with no differences in accuracy across trial types (*F*_(2,154)_ = 0.679, *p* = 0.508; Fig. 1*E*). The false alarm rate, defined as PM target responses on probes where the target was not present, was low (mean = 0.60% of probes, 95% CI = −0.72% to 1.92%). Because PM accuracy was stable across the dynamic trial types (increasing- and decreasing-difficulty trials), and OG accuracy was not impacted by the presence of the PM task on these trials, we concluded that participants were not sacrificing accuracy on one task to perform the other. Therefore, any RT differences on the OG task during PM trials could reasonably be interpreted as reflecting differences in strategy used to perform the PM task, rather than a speed/accuracy trade-off between the dual tasks.

### Linking shifts in PM strategy to PM performance

We hypothesized that not only would individuals demonstrate gradual shifts in PM strategy in response to changing task demands, but that changes in PM strategy would be related to PM performance. Based off of the dual methods of control (DMC) framework (Braver, 2012) and dynamic multiprocess view of prospective memory (Scullin et al., 2013), we reasoned that on decreasing- and fixed-difficulty trials, when the resources to implement proactive control became readily available, proactive control would benefit PM performance. However, on increasing-difficulty trials, ongoing task demands make it difficult to implement the proactive control mechanisms of strategic monitoring and/or sustained representation of the PM target. Therefore, individuals may benefit from shifts toward reactive control strategies on these trials, as they attempt to preserve performance on the PM task in the face of increasing demands. To evaluate this hypothesis, we tested the relationship between PM cost slope and PM accuracy across all trial types.

We used bootstrapped logistic regression to relate these two measures separately for increasing and decreasing trials (Fig. 2*F*). On decreasing trials, larger positive PM cost slopes (reflecting a shift toward proactive control) were related to better PM performance (β_dec_= 0.018, *p* < 0.001, 95% CI = 0.008–0.028). On increasing trials, larger negative PM cost slopes (reflecting a shift toward reactive control) were numerically related to better PM performance, but this relationship did not reach statistical significance (β_inc_= −0.006, *p* = 0.119, 95% CI = −0.015 to 0.004). Critically, there was an interaction of PM cost slope and trial type on PM accuracy, with the direction of PM cost slope leading to different consequences on increasing versus decreasing trials (β_interaction_ = 0.024, *p* < 0.001, 95% CI = 0.011–0.037). On fixed trials (data not shown), the relationship between PM cost slope and PM accuracy was positive (β_fix_ = 0.020, *p* = 0.003, 95% CI = 0.006–0.033), which was similar to the situation in decreasing trials (β_diff_ = 0.001, *p* = 0.423, 95% CI = −0.015 to 0.017), but was significantly more positive than on increasing trials (β_diff_ = 0.025, *p* = 0.001, 95% CI = 0.009–0.041).

The relationship between PM cost slope and PM accuracy survived after controlling for other possible explanatory variables. We first compared the by-trial AIC- and variance-explained (*R* ^2^) values for predicting PM accuracy on increasing and decreasing trials using the following five different factors: average OG RT, average OG accuracy, OG RT slope, average PM cost, and PM cost slope. We ran a bootstrap analysis (*n* = 3000 iterations) comparing models that included the interaction of trial direction and each of these factors for predicting PM accuracy, and we extracted AIC and *R* ^2^ scores from each iteration. The AIC scores were converted to Akaike weights (Wagenmakers and Farrell, 2004) for comparison across models. The model using PM cost slope as a predictor of PM accuracy had a significantly higher Akaike weight (mean = 0.99, SE = 0.001) than any other model (all other means, <0.01). The *R* ^2^ value for the model containing only PM cost slope was also the highest (mean *R* ^2^ value: PM cost slope = 0.08, average PM cost = 0.03, average OG RT = 0.03, average OG Acc = 0.005, OG RT slope = 0.005).

To see whether this relationship held at the subject level, we also performed partial regressions comparing how much PM cost slope predicted PM accuracy while controlling for the other variables. After controlling for average OG RT, average OG accuracy, average PM cost, and OG RT slope one at a time, PM cost slope still explained a significant proportion of variation in PM accuracy (*R* ^2^ = 0.09, *p* = 0.009; *R* ^2^ = 0.12, *p* = 0.002; *R* ^2^ = 0.12, *p* = 0.001; *R* ^2^ = 0.12, *p* = 0.002; respectively).

This same by-trial relationship between PM cost slope and PM accuracy existed across participants as well. Participants who on average showed larger shifts toward proactive control (more positive PM cost slopes) benefited more on decreasing trials, and participants who showed larger shifts toward reactive control (more negative PM cost slopes) benefited more on increasing trials (β_interaction_ = −0.018, *p* = 0.002, 95% CI = −0.013 to −0.024; data not shown). In summary, these results provide behavioral evidence that individuals shifted PM strategy from moment to moment in response to changing OG task demands. These shifts in cognitive control were adaptive because their direction and magnitude were related to successful PM performance.

### Neural measures of PM intentions

For the participants who performed this task in the MRI scanner (*N* = 28), we evaluated whether a neural measure of PM intention-related brain activity (Lewis-Peacock et al., 2016) could provide additional insight into the link between PM strategy selection and memory performance. First, we identified regions that were significantly engaged by the PM task above and beyond the OG task in isolation (Fig. 3*A*). These regions are consistent with previous literature on PM intention maintenance (McDaniel et al., 2013; Beck et al., 2014; Cona et al., 2015; Lewis-Peacock et al., 2016). From these brain regions, fMRI pattern classifiers were used to quantify the degree of PM intention-related processing across each trial. The strength of PM intention processing was operationalized as the difference in classifier evidence for the PM-relevant category versus the PM-irrelevant category (e.g., “face minus scene” for a face-target PM trial). Trained classifiers performed well above chance at predicting the PM target category of the current trial (classifier AUC: for faces: 86.94, SE = 0.01; for scenes = 88.54, SE = 0.01; for non-PM trials: 83.76, SE = 0.01; and for rest = 99.62, SE = 4.7 * 10^−4^). Across trials, this neural measure varied systematically with OG task difficulty (Fig. 3*B*). The neural evidence of PM intention processing decreased as task demands increased (β = −0.005, *p* < 0.001, 95% CI = −0.008 to −0.002, marginal *r* ^2^ = 0.039).

Table 3-1 **PM > Non-PM GLM Analysis.** FSL’s FEAT was used to identify voxels that were more responsive on PM trials than on non-PM trials (cluster correction, p < .001). This table lists the coordinates and descriptors for all significant voxel clusters. Download Table 3-1, DOCX file.

10.1523/ENEURO.0250-19.2019.f3-2 Figure 3-2 **Surface searchlight analysis.** Results from the surface-based searchlight classification analysis to decode the PM intention on PM trials. Vertices in red indicate those that survived threshold-free cluster enhancement significance testing (H_0_ mean = 50%, p<.001). Results indicate that classification was successful only in two particular posterior regions: the ventral temporal cortex and lateral occipital cortex. Notably absent from this map is the anterior lateral prefrontal cortex. To perform this analysis, anatomical surface outputs from Freesurfer *recon-all* were converted to AFNI/SUMA format using *SUMA_Make_Spec_FS*. Surfaces were remapped to a standard topology using *MapIcosahedron* and co-registered to a reference functional volume using *align_epi_anat* so that functional data could be masked by the surface volume. Voxels determined to not be part of the surface were masked out of the searchlight analysis. Surface searchlight analysis was performed in MATLAB using functions from the CosMoMVPA toolbox. Each searchlight sphere was determined by selecting the 100 closest vertices to a center vertex according to geodesic distance. L2-weighted logistic regression classifiers were trained on four categories and tested within each searchlight sphere using a k-fold cross validation procedure. Only the five main PM task blocks were used for this analysis, and data from the localizers were excluded. That meant that on each k-fold iteration, 4 out of 5 PM-task blocks were used for training the classifier, and one held out block was used for testing. Accuracy was then computed on face and scene probes. The face/scene accuracy across all five folds was averaged and that value was assigned to the center vertex of that sphere. Download Figure 3-2, DOCX file.

However, within trials the neural measure of PM intention processing did not vary systematically across time points (mean slope of PM EV = 0.007 EV/s, 95% CI = −0.002 to 0.016, *t*_(27)_ = 1.57, *p* = 0.128). There were also no differences in by-trial PM intention evidence slopes across increasing, decreasing, and fixed-difficulty trials (*F*_(2,54)_ = 1.35, *p* = 0.269). The stable level of PM intention processing over the course of a single trial may be a measurement limitation due to the temporal sluggishness of the BOLD signal. Alternatively, it could also reflect the engagement of a prospective “retrieval mode” (Guynn, 2003, 2008; Cona et al., 2014), which has been described as a more sustained and relatively inflexible component of proactive control that involves PM items being held in some prioritized state of working memory (Underwood et al., 2015). Therefore, we computed the average classifier evidence for the PM intention on each trial and related this (rather than the slope) to PM accuracy. A mixed-effect ANOVA found that there were no overall differences in average PM intention evidence across trial types (*F*_(2,54)_ = 0.40, *p* = 0.670). This result was expected because increasing and decreasing trials spanned the same range of difficulty levels (e.g., 1–15 vs 15–1). We found that average PM intention evidence correlated positively with PM accuracy on decreasing trials (β_dec_ = 0.754, *p* = 0.017, 95% CI = 0.047–1.50; Fig. 3*C*, red) and fixed trials (β_fix_ = 0.976, *p* = 0.039, 95% CI = −0.121 to 2.062; data not shown), but not on increasing trials (β_inc_ = 0.280, *p* = 0.255, 95% CI = −0.679 to 1.123; Fig. 3*C*, blue). There were no reliable differences in this statistic between increasing trials and either decreasing trials (β_interaction_= 0.474, *p* = 0.199, 95% CI = −0.627 to 1.623) or fixed trials (β_interaction_ = 0.223, *p* = 0.361, 95% CI = −1.119 to 1.473).

### Combining behavioral and neural measures to predict PM performance

**Figure 4.**
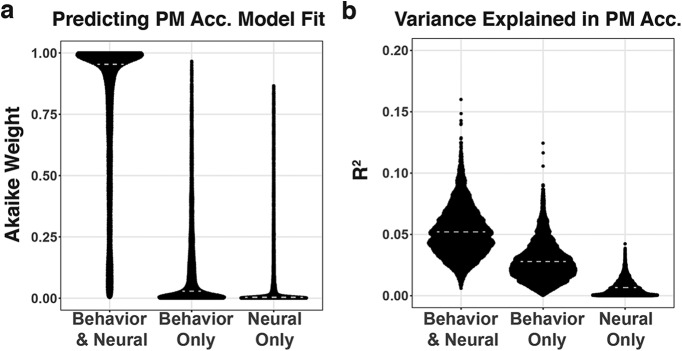
Model comparisons using behavioral and neural metrics to predict PM performance. Model weights and *R* ^2^ values were computed across bootstrap iterations (*n* = 10,000) to test model differences. ***a***, wAICs across bootstrap iterations for each model. ***b***, Explanatory power of each model (*R* ^2^) shown as distributions across bootstraps. Medians are indicated by dashed gray lines.

We sought to test whether combining both the time-sensitive but indirect behavioral metric of PM cost slope (putatively reflecting dynamic shifts in PM strategy) and the coarser but more direct neural metric of PM intention evidence (putatively reflecting sustained PM engagement) could improve our prediction of PM accuracy on a trial-by-trial basis. There was no by-trial correlation between these measures (mean *r* = 0.02, 95% CI = −0.36 to 0.39, *p* = 0.92), suggesting that the two metrics could provide unique information about task performance. We performed a bootstrap analysis to calculate the AIC values for models predicting PM accuracy, including all possible combinations of the predictors PM cost slope, PM intention evidence, and trial type (increasing/decreasing). We then selected the best performing model that included (1) a neural and a behavioral metric, (2) only a behavioral metric, or (3) only a neural metric. Next, we converted AIC scores for these three models to wAIC values, allowing us to directly compare AIC values as conditional probabilities (Wagenmakers and Farrell, 2004). The results show that the combined Behavior and Neural model (i.e., the full model including all main effects; all two-way interactions; and the three-way interaction of PM cost slope, PM intention state, and trial direction) was the best model (Wilcoxon median Akaike weight = 0.889, Wilcoxon 95% CI = 0.883–0.896, *p* < 0.001; Fig. 4*A*). This combined model was significantly more likely than either the best Behavior-Only model (Wilcoxon median ratio = 149.91, Wilcoxon test, 95% CI = 135.47–165.63, *p* < 0.001) or the best Neural-Only model (Wilcoxon median ratio = 4.5 * 10^9^, Wilcoxon test, 95% CI = 3.9 * 10^9^ to 5.2 * 10^9^, *p* < 0.001).

The Behavioral and Neural model explained the most variance in PM accuracy, with an *R* ^2^ value of approximately double that of the best Behavior-Only model (*R* ^2^ = 0.052 vs 0.028; Fig. 4*B*). Having found that the full Behavioral and Neural model was the best predictor of PM performance, we further investigated the reliability of each relationship in the combined model using the bootstrap approach. In this analysis, we were also interested in whether the relationships we found independently between PM accuracy and PM cost slope and then between PM accuracy and PM intention evidence would be qualified by any reliable interactions in the full model. We found that, although including the three-way interaction term and multiple two-way interaction terms resulted in the lowest overall AIC score, the only statistically reliable interaction was that of PM cost slope and trial direction (*p* = 0.026), confirming that the analysis shown in Figure 2*F* holds in a more comprehensive model. Additionally, we found that PM intention evidence had a reliably positive relationship to PM accuracy on decreasing-difficulty trials (mean = 0.751, *p* = 0.019), but that relationship was not reliable on increasing-difficulty trials (mean = 0.284, *p* = 0.257), also confirming the analysis shown in Figure 3*C* holds in a more comprehensive model. In summary, our model tests revealed the following three main results: (1) including both the neural and behavioral metrics of proactive control improved prediction of PM accuracy over using either metric independently; (2) PM cost slope was differentially predictive of PM accuracy for increasing versus decreasing trials, replicating the relationship from our behavior-only analysis above; and (3) higher levels of PM intention evidence were positively related to PM performance on decreasing-difficulty trials, but there was no reliable relationship on increasing-difficulty trials.

## Discussion

This study investigated how navigating an environment with rapidly shifting cognitive demands impacts how we remember to perform future actions. A delayed-recognition PM task was combined with a dynamic visual search OG task that varied in difficulty from moment to moment. When task difficulty was low, there was greater behavioral interference from the PM task (PM cost: slower RTs in the OG task) and stronger neural representation of the PM intention (PM intention evidence: classifier evidence for the PM target category in PM-sensitive brain regions). Both of these measures reflect components of proactive control (Braver, 2012) and were negatively correlated with OG task difficulty. The behavioral measure varied within a trial according to the task demands, whereas the neural measurement was stable within a given trial but varied across trials. Combining these behavioral and neural measures provided the best prediction of PM accuracy from trial to trial. Together, these results suggest that individuals dynamically adjust their PM strategy in response to changes in environmental demands. Critically, we found that these shifts in PM strategy were adaptive because greater shifts (in the appropriate direction toward proactive or reactive control) were related to improvements in PM performance. The present results demonstrate that the ability to flexibly adjust cognitive control strategies, in response to changes in environmental demands, is an important contributor to successful execution of delayed intentions.

We computed the following two distinct metrics of proactive strategy use: a time-sensitive behavioral measure of PM cost, and a more tonic neural measure of PM intention processing. The amount of PM costs (the behavioral measure) has been repeatedly linked to levels of strategic monitoring for the PM intention (Smith, 2003; Einstein and McDaniel, 2005). Here, we found that changes in the amount of PM costs over the course of a trial were associated with better performance (Fig. 2*F*). The DMC framework suggests that proactive control would be favored on decreasing-difficulty trials, when the OG task becomes progressively easier, because attention and working memory resources should be readily available to accomplish both the OG task and PM task successfully. On these trials, strategically monitoring for the PM intention may be worth the extra cost incurred in RTs on the OG task. Our results support this idea, showing that when participants reallocated cognitive resources to use proactive control on the PM task (positive PM cost slopes within a trial), PM performance improved.

However, we found that in increasing-difficulty trials, there was an opposite relationship between PM cost slope and PM accuracy, where larger PM cost slopes were related to moderately worse PM performance. The DMC framework predicts that as difficulty increases, the ability to strategically monitor for the PM intention can be compromised, and reliance on proactive control may lead to deficits in PM performance. Such deficits may arise from interference in working memory caused by failed attempts to maintain a robust representation of the PM target in the presence of distractors, a reduced ability to shift attention between the two tasks to strategically monitor for the PM cue effectively, or a reduced ability to perform the PM intention even after noticing a prospective cue (West et al., 2007a; Zuber et al., 2016; Ballhausen et al., 2017). Consistent with these ideas, we found that when participants attempted to maintain high levels of proactive control even as the OG task difficulty increased (i.e., PM cost slopes were positive on these trials), PM performance suffered. The relationship between PM cost slope and PM accuracy on increasing-difficulty trials suggests that reactive control can be used successfully in situations that are not well suited for proactive control (e.g., under high cognitive load). The results from this study build on previous research that demonstrated in some circumstances there is a benefit to using proactive control (Smith 2003; Shelton and Scullin, 2016), as we found on decreasing- and fixed-difficulty trials, and some circumstances where there is no benefit (Einstein and McDaniel, 2005; Scullin et al., 2010a,b), as we found on increasing-difficulty trials.

In our study, participants knew that a PM target would reappear relatively soon after it was introduced (between 2 and 30 s later with 100% fidelity). The DMPV framework (Scullin et al., 2013; Shelton and Scullin, 2017) posits that in contexts similar to our experiment, where PM occurrences are highly probable, individuals are biased toward and benefit from using proactive control. On trials with fixed difficulty, we found a consistent increase in PM costs across each trial (positive PM cost slopes), and greater increases in cost were related to better PM performance. This indicates a beneficial, perhaps default, shift toward proactive control in this paradigm for which there is an increasing probability of a PM event throughout each trial. Shifts toward proactive control were even stronger (and beneficial for performance) on decreasing-difficulty trials as more cognitive resources became available over time. This result is in line with previous work showing that, given the available resources, individuals will increase monitoring as the expectancy of the PM event increases, and that increased monitoring is beneficial to PM performance (Kuhlmann and Rummel, 2014; Loft, et al., 2014; Bowden et al., 2017).

Additionally, although overall PM accuracy on increasing and decreasing trials was equivalent, the range of accuracies differed between trial types. On decreasing-difficulty trials, participants performed the PM task dramatically better when shifting toward proactive control and worse when shifting toward reactive control. On increasing-difficulty trials, while this relationship was numerically reversed, the difference in performance across strategy types was reduced (Fig. 2*F*). In other words, on decreasing-difficulty trials there was a clear and large benefit to PM performance when PM cost slopes were positive, while on increasing-difficulty trials PM performance was more similar in respect to PM strategy. Additionally, when collapsing across all trials, we found a small performance advantage to using proactive control. On trials when the PM target appeared while PM costs were high, detection accuracy of the PM target was high (mean = 76.62%, SEM = 1.94%, average *N* = 37.7 trials/participant). On trials where PM costs were absent (indicating no evidence of proactive control) when the PM target appeared, accuracy was worse (*t*_(76)_ = 4.684, *p* < 0.001), but still relatively good and well above floor (mean = 67.81%, SEM = 2.67%, *N* = 12.1 trials/participant). The behavioral data from our study suggest that over short, highly predictable intervals (<30 s), proactive control is the more reliable strategy for PM intention fulfillment, but only when monitoring for and maintenance of a PM intention can be adequately performed. In other circumstances, such as those with high concurrent demands, individuals benefit from offloading the PM task to reactive control in the form of equal performance with less cost. Future work should investigate the impact of strategy flexibility across longer delays between encoding and retrieval of intentions, and whether a proactive benefit may still be observed.

Many models of PM have focused on the relationship between proactive and reactive control. Some studies (Gilbert et al., 2013) propose a central executive process that allocates resources toward either proactive or reactive control along a continuum, or strikes some balance between attention and external stimuli versus internal stimuli (Cona et al., 2015). The two-component model by Guynn (2003, 2008) of proactive control dissociates a flexible, strategic monitoring component from a more tonic component, referred to as the “PM-retrieval mode,” which involves sustained maintenance of the PM task set. This second component is described as load invariant and relatively inflexible, while the first component is thought to be dynamically sensitive to environmental factors (Underwood et al., 2015). Recent work has suggested that the PM-retrieval mode is also able to be strategically adjusted to some extent (Whitehead and Egner, 2018), but the amount of cognitive resources needed to maintain it can negatively affect monitoring ability (Ballhausen et al., 2017).

In the present study, we propose that the behavioral measure of PM cost reflects the strategic monitoring component of this model, whereas the neural measure of PM intention evidence reflects the PM-retrieval mode component. Our results implicated neural regions commonly associated with working memory (Eriksson et al., 2015; Extended Data Table 3-1, specifics) in supporting PM performance. We found that on decreasing-difficulty trials (which, according to the behavioral analysis, favor a shift toward proactive control), PM intention maintenance was positively correlated with memory performance, but on increasing trials (which favor a shift toward reactive control) it was not (Fig. 3*C*). Although individuals sometimes exhibited a high level of PM readiness, this did not influence task performance in situations that favored reactive control. These results are again in line with previous research suggesting that PM intention maintenance could be beneficial to PM performance in some situations but was not necessary for successful realization of PM intentions in all situations (Cona et al., 2014).

We find that our behavioral and neural metrics provide complementary but independent information about PM performance, approximately doubling the predictive power of our model when the neural measure of PM intention maintenance was combined with the behavioral metric of PM cost slope (Fig. 4*B*). This result suggests that our measures are capturing different components of proactive control, though the current design does not specify the relative contribution of strategic monitoring versus PM intention maintenance. A future direction will be to use more time-sensitive neural measures like EEG (electroencephalogram) and eye-tracking to measure the active maintenance of PM intentions in dynamic environments, as well as to better identify late-retrieval mechanisms that are characteristic of reactive control strategy use.

Contrary to previous studies that have indicated a key role for the anterior prefrontal cortex (aPFC) in representing prospective intentions (Gilbert, 2011; Momennejad and Haynes, 2012, 2013), we found that regions known to support perception and working memory for the PM intentions used here [i.e., the ventral temporal cortex (Grill-Spector and Weiner, 2014; D’Esposito and Postle, 2015) for face and scene stimuli] were most important for identifying PM intention maintenance (as identified by a surface-based searchlight analysis; Kriegeskorte et al., 2006; Oosterhof et al., 2016; Extended Data Fig. 3-2). The searchlight results suggest that PM item retrieval is primarily mediated by these more posterior regions, while the prefrontal region may have a more abstracted involvement, like PM state or rule maintenance (i.e., maintaining whether or not one has a prospective intention at the moment). However, the lack of above chance PM intention decoding in the PFC may be related to a lower signal-to-noise ratio or due to the “mixed selectivity” of prefrontal neurons (Bhandari et al., 2018). Further research is needed to better understand how and where PM intentions are represented and the specific role of classically identified regions like the anterior prefrontal cortex.

Our results are consistent with the dual mechanisms of cognitive control framework (Braver et al., 2007; Braver, 2012) and the dynamic multiprocess view of PM (Scullin et al., 2013; Shelton and Scullin, 2017). Both dual-mechanism frameworks posit that individuals can use two different methods of cognitive control to fulfill prospective intentions, and that they can flexibly adjust their control strategy in response to environmental factors, such as cognitive load or PM target expectancy. However, neither framework formally describes whether that adjustment is a fluid process or an all-or-none “switch” between strategies. Here, our evidence suggests that there are graded levels of control between proactive and reactive strategies that people engage along a continuum.

An alternative explanation for the present results is that shifts in PM cost may not reflect shifts between proactive and reactive control strategies per se, but rather shifts between stronger and weaker levels of proactive control. Unitary models of PM such as the preparatory attention and memory “PAM” theory (Smith, 2003, p. 200) propose that successfully fulfilling prospective intentions relies on some level of proactive preparation in all situations. However, we believe this interpretation of our data are less likely than the dual-mechanisms account. According to the PAM model, we should expect extremely poor PM performance when evidence of proactive control is absent, however this was not the case. As mentioned previously, performance on trials where there were no observed costs on end probes (*n* − 3 to *n* − 1 before PM − target), PM accuracy was still higher than the PAM model would predict (mean = 67.81%, SEM = 2.67%). PM performance was also strong on trials where the neural measure of PM intention evidence was absent (mean = 81.1%, SE = 3.2%, average *N* = 11.5 trials/participant), and also on trials where both PM cost and PM intention evidence were absent (mean = 70.5%, SE = 6.5%, average *N* = 2.8 trials/participant). Incidentally, the link between PM cost and PM accuracy in the present study closely replicates prior work using a similar, though static, task design (Lewis-Peacock et al., 2016). In that experiment, the researchers found that on blocks where participants were biased toward reactive control, PM accuracy was 66.0% (SEM = 4.1%), and when participants were biased toward proactive control, PM accuracy was 71.2% (SEM = 3.0%).

One limitation of the current study is its reliance on the behavioral PM cost measure to infer PM strategy use. While this has become a standard approach, PM cost is nonetheless an indirect measure of PM strategy, the underlying source of which is still under debate (Boywitt and Rummel, 2012; Ball et al., 2015; Heathcote et al., 2015; Strickland et al., 2017). We sought to complement this indirect measure with a more direct measure of PM intention processing using fMRI pattern classifiers to track PM intention maintenance. However, fMRI is sluggish and not ideal to observe time-sensitive shifts in neural coding. It is possible that our neural measure incorporates aspects of both PM intention maintenance as well as strategic monitoring. However, these neural measures were not correlated with PM cost slopes, which are believed to reflect changes in monitoring. In addition, previous work has found that in contexts where participants are biased toward reactive control, the level of monitoring is not related to PM performance (Harrison and Einstein, 2010; Loft et al., 2014); however, it is related to PM performance when participants are biased toward proactive control (Loft et al., 2014; Ball et al., 2015; Lewis-Peacock et al., 2016).

In conclusion, we developed a novel dual-task paradigm to show that people solve prospective memory problems by flexibly shifting between proactive control and reactive control in response to changes in ongoing cognitive demands. We found evidence for two different components of proactive control—strategic monitoring, measured behaviorally, and PM intention maintenance, measured neurally—which independently fluctuate and contribute to PM performance. These shifts were adaptive in that adjustments of control toward the strategy favored for a given situation (e.g., shifting toward proactive control when demands decreased across time) led to better PM performance. These results extend dual mechanism accounts of PM by demonstrating that cognitive flexibility (i.e., adapting cognitive control strategies to the environment) is beneficial for remembering to perform future intentions.

## Synthesis

Reviewing Editor: Morgan Barense, University of Toronto Decisions are customarily a result of the Reviewing Editor and the peer reviewers coming together and discussing their recommendations until a consensus is reached. When revisions are invited, a fact-based synthesis statement explaining their decision and outlining what is needed to prepare a revision will be listed below. The following reviewer(s) agreed to reveal their identity: Sam Gilbert, Derek Nee.

We were all in agreement that the manuscript provides novel insight into the cognitive and neural mechanisms governing prospective memory, and we were all impressed with the elegance of the design and the rigor of the analytic approach. We believe that conclusions are important for the field. Each reviewer provided specific feedback (provided below), which I hope will be helpful as you prepare the revision.

REVIEWER 1:

This is an interesting manuscript which I think can make a helpful addition to the field. In prospective memory research, terms like ‘strategic monitoring’ or ‘proactive control’ are often used but they are poorly defined. This work helps to characterise the nature of these processes and can form a useful basis for future work investigating their computational properties.

I think the manuscript provides two main advances:

- it provides empirical evidence addressing the question of whether strategy choices in prospective memory tasks should be interpreted in terms of binary decisions between strategy alternatives, or as reflecting a graded continuum. Results point towards the latter interpretation, which is interesting seeing as it contrasts with some views in the literature (e.g. Smith's multinomial model, which sees monitoring as an all-or-nothing process

- it finds that behavioural and neural measures of proactive control independently predict prospective memory accuracy. This suggests that including neural measures of intention-related processing provides additional information beyond behavioural measures alone

Having said that, I did find the manuscript hard to follow at points and I think the authors could do more to clarify the key questions they are asking, why their design is relevant to those questions, and how the analyses they perform adjudicate between the possibilities. For example, the abstract states “it is unclear if and how people update their strategy when the situation changes”. Regarding the “if” question, there is abundant behavioural evidence from the prospective memory field that people adjust their strategy in response to different sorts of manipulations such as task importance or “cue focality”. So I'm not sure what the authors are saying is unclear. Regarding the “how” question, while the evidence produced by the authors is very interesting in showing how different types of trial are associated with behavioural and neural markers of different strategies, I'm not sure how this work tells us “how” the strategy shifts occur. Overall, I think the paper could be improved by a clearer statement towards the beginning of the main theoretical questions and exactly how the experiment addresses them. I think this would be particularly helpful in advance of the section titled “Linking Shifts in PM Strategy to PM Performance” (pp. 23-25) which is very hard to follow otherwise.

I also think that the authors could be a bit clearer about their conception of the relationship between proactive and reactive control. They argue that as the task becomes progressively more difficult, it makes sense to shift towards a reactive control strategy because a reliance on proactive control would be harmful to performance. But if a reactive control strategy is sufficient, why not just use this strategy all the time and avoid the costs associated with the proactive strategy? In other words, if a reactive strategy is sufficient, why not employ this for all trials, rather than shifting to it only when the ongoing task becomes more difficult? The behavioural data shows that as the ongoing task becomes more difficult, the PM cost is reduced (suggesting reduced proactive control) with no corresponding decrease in PM accuracy (“PM Accuracy was equivalent across difficulties”; p. 16). In that case, why engage a costly proactive strategy at all?

There is an interesting analysis reported on p. 21 of whether strategy effects should be seen as binary or graded. This is based on a model selection approach in which the graded model was preferred. However, seeing as this was based on regressions conducted on single-trial data, any binary strategy shift would be hard to detect amongst the noise. Given that the graded model had fewer free parameters, might this model have been selected simply because the noisy evidence for a binary strategy shift was too weak to overcome the penalty associated with the additional parameters?

REVIWER 2:

This study examined cognitive and neural mechanisms underlying prospective memory. In particular, the authors examined how changes in the difficulty of an ongoing task affected prospective memory. The authors adopted a proactive/reactive control framework in which they hypothesized that ongoing demands would cause shifts is the use of proactive versus reactive control mechanisms. Explicitly, easier ongoing demands should allow for more proactive control, whereas difficult ongoing demands would require reactive control. Behaviorally, proactive control was indexed by the degree to which performing an ongoing task concurrently with prospective memory demands effected an increase in RT relative to performing the ongoing task in isolation. Neurally, proactive control was indexed by the degree to which a machine learning classifier found evidence for category-specific activity related to the prospective memory target.

Consistent with their hypothesis, the authors found that dual-tasking costs diminished with difficulty. Moreover, if the ongoing task was easier over time, dual-tasking costs increased, which the authors interpreted as engagement of proactive control. These same costs decreased if the ongoing task was harder over time, which the authors interpreted as reactive control. Increased dual-tasking costs as ongoing demands decreased were associated with better prospective memory performance, consistent with these costs reflecting proactive control. Furthermore, increased neural evidence of category-specific activity was also associated with improved prospective memory performance, again consistent with the idea that these indices reflect proactive control. Interestingly, behavioral and neural metrics of proactive control were unassociated and made independent contributions to prospective memory performance, potentially reflecting dynamic and tonic contributions, respectively.

Overall, this is an excellent manuscript. The experimental design is elegant and powerful. The analytic approach is superb. The results and conclusions are impactful. Hence, I am enthusiastic about this study. As a result, my concerns are only minor.

Minor Concerns

1) Although the data on decreasing and fixed trials are clear and consistent with the authors' framework, I found the data on the increasing trials difficult to reconcile with the hypothesis. The idea that shifts towards reactive control would improve *prospective memory* simply does not make sense to me. If participants are shifted towards reactive control, the natural result that seems to follow from the cited work is that prospective memory performance should suffer in order to sustain performance on the ongoing task. The authors find a trend towards the opposite using the behavioral cost slope, and a flat trend using the neural evidence. I suspect that these data indicate that a different mechanism is at play here.

Given that the relationship between neural evidence and prospective memory performance is flat on increasing trials, this would suggest that participants are not more reactive on the increasing trials. Instead, the reduction of the dual-tasking cost may reflect a sort of slack logic or parallel processing account (c.f. Pashler, 1984; Rubinstein et al., 2001). That is, if we assume that searching for the arrow target can be done partially in parallel with sustaining/searching for the prospective memory target, the increased search RTs provide an opportunity to complete the prospective portion of processing before the arrow search completes. Hence, although participants may get more proactive over time in the increasing trials, that proactive processing is not getting reflected in the RT due to the partial parallelism.

If we assume that dual-tasking costs are then absorbed into the overall RT during the increasing trials, relatively long trial RTs may reflect poorer task engagement, which is then related to decreased prospective memory performance. That is, the long RT increasing trials reflect zoning out more so than proactive control. One way to test this idea would be to examine the relative recruitment of PM-related areas and task-negative (e.g. default) areas as a function of PM cost slope and trial type (increasing vs decreasing). The prediction based on this account would be that increased PM cost slopes on the increasing trials are associated with greater engagement of task-negative areas. This would be an interesting and surprising result given that increased RTs are generally associated with more task-positive engagement. If this turns out, then the authors would have two distinct neural indices (PM evidence, task-negative recruitment) that potentially independently contribute to the waxing and waning of control in this paradigm.

2) For the most part, the authors have done an excellent job in explaining a number of challenging topics, from the cognitive model, to the paradigm, to the analyses. However, there are a few areas that could use touching up:

A) The bootstrapped logistic regression was something that I have not come across often within this literature. It would be useful to add in references that support the legitimacy of this procedure. In particular, it is unclear how sampling from the sample lends inference to the population. It seems to me, this affords only inferences to the sample itself.

B) More explicit details of how the classifier evidence value is computed and how AUC is computed are needed. The release of the code will certainly help to clarify things, but the average reader would probably want greater clarity without having to sift through the code.

C) On page. 29, I lost the thread trying to parse the two- and three-way interactions. I think these are mostly re-descriptions of what has already been documented in Figures 2 and 3, but I wasn't sure.

D) On page 32 “Consistent with these ideas, we found that when participants did not divert cognitive resources away from the PM task as OG task difficulty increased, PM performance suffered.” This odd conclusion is at the heart of issue 1 above. My understanding is that if participants do not divert resources away from the PM task, the OG task should suffer, not the PM task. If I have misunderstood, the idea requires more clarification.

To close, I thoroughly enjoyed this manuscript and look forward to its publication.
