# Supplementary material for: Cognitive Flexibility Improves Memory for Delayed Intentions
Source: eNeuro. 2019 Nov 1;6(6):ENEURO.0250-19.2019. doi: 10.1523/ENEURO.0250-19.2019 (PMC6838690; doi:10.1523/ENEURO.0250-19.2019)
Supplement: Figure 1-1 — Average reaction time (y-axis left, blue) and accuracy (y-axis right, red) for pilot participants are plotted across each difficulty level of the ongoing visual search task. Reaction time increases and accuracy decreases from the easiest difficulty (1) to the hardest (14). The purpose of the first behavioral pilot study was to determine if the ongoing task could be parametrically modulated in a controlled manner. For this pilot study, participants (n = 15) performed the ongoing task in isolation. On each probe, participants indicated the absence or presence of the arrow target (rightward facing horizontal arrow) in a newly generated visual-search array (every 2 s) with a button press (left: absent; right: present; response deadline: 1.9 s). Target arrow location was counterbalanced between the top and bottom half of the screen. Non-target arrows appeared in set positions around the circular array, oriented within some distribution of angles determined by the current task difficulty setting. Participants sat approximately 18 in. away from the screen, and all 10 arrows, which were .64° by .22° in shape, were 3.18° away from the center of the screen. OG task difficulty was manipulated on each probe by adjusting two parameters controlling the orientation of the distractor arrows: their minimum similarity to the target and their similarity to other distractors. For distractor-to-target similarity, a minimum angular distance for distractors from the target (i.e., horizontal or 0°) was set to either 5, 15, 25, 45, or 65 degrees. For distractor-to-distractor similarity, the maximum variance from the minimum angular distance was set to either 10, 20, or 40 degrees. The factorial combination of these parameters (excluding any combination where minimum plus variance could exceed the 90° vertical plane) created 14 difficulty conditions. Participants performed three blocks of trials separated by short voluntary breaks for rest. Each block contained 14 mini-blocks comprised of 20 [file sup_enu-eN-NWR-0250-19-s01.docx]

**Extended data for:**Cognitive flexibility improves memory for delayed intentions

**Figure 1-1.** Average reaction time (y-axis left, blue) and accuracy (y-axis right, red) for pilot participants are plotted across each difficulty level of the ongoing visual search task. Reaction time increases and accuracy decreases from the easiest difficulty (1) to the hardest (14). The purpose of the first behavioral pilot study was to determine if the ongoing task could be parametrically modulated in a controlled manner. For this pilot study, participants (n = 15) performed the ongoing task in isolation. On each probe, participants indicated the absence or presence of the arrow target (rightward facing horizontal arrow) in a newly generated visual-search array (every 2 s) with a button press (left: absent; right: present; response deadline: 1.9 s). Target arrow location was counterbalanced between the top and bottom half of the screen. Non-target arrows appeared in set positions around the circular array, oriented within some distribution of angles determined by the current task difficulty setting. Participants sat approximately 18 in. away from the screen, and all 10 arrows, which were .64° by .22° in shape, were 3.18° away from the center of the screen. OG task difficulty was manipulated on each probe by adjusting two parameters controlling the orientation of the distractor arrows: their minimum similarity to the target and their similarity to other distractors. For distractor-to-target similarity, a minimum angular distance for distractors from the target (i.e., horizontal or 0°) was set to either 5, 15, 25, 45, or 65 degrees. For distractor-to-distractor similarity, the maximum variance from the minimum angular distance was set to either 10, 20, or 40 degrees. The factorial combination of these parameters (excluding any combination where minimum plus variance could exceed the 90° vertical plane) created 14 difficulty conditions. Participants performed three blocks of trials separated by short voluntary breaks for rest. Each block contained 14 mini-blocks comprised of 20 visual-search trials of one difficulty level. Difficulty level was pseudo-randomly selected, with the only limitation being that each of the 14 difficulty levels was selected exactly three times. As difficulty increased, accuracy decreased (F(13,182)=74.89, p<.001) and reaction time increased (F(13,182)=39.53, p<.001).

**Figure 1-2.** The purpose of the second behavioral pilot study was to determine if PM strategy (as measured by PM cost) could be modulated by the difficulty of the ongoing task. The task design for this study was nearly identical to that used in the main experiment, but here OG task difficulty was held constant as either *easy* (difficulty level 4) or *hard* (difficulty level 12) for the entirety of each block and across each trial. Participants (n = 20) completed one block (15 trials per block) at each difficulty level. **a)** PM cost was calculated by subtracting the average non-PM trial OG RT from the average PM trial OG RT at each difficulty level for each participant. The distribution and median are represented. PM cost significantly varied as a function of OG task difficulty (F(1,19) = 35.63, p<.001), with cost being higher at the easier difficulty (M = 0.134 s (SE = 0.012)) than at the harder difficulty (M = 0.031 s (SE = 0.012)). * p<.05. **b)** PM accuracy was calculated at each difficulty for each participant. The distribution and median are represented. PM accuracy was equivalent across conditions (F(1,19) = 0.785, p = 0.387; easy = 71.0% (4.5%), hard = 64.5% (5.8%)).


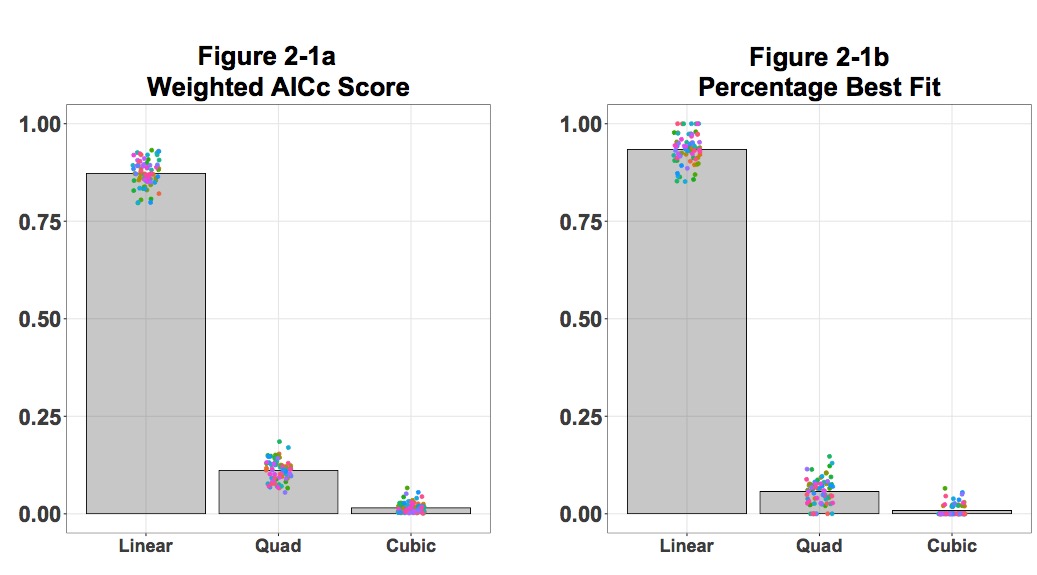


**Figure 2-1.** Visualization of model comparisons for by-trial estimates of PM strategy shifts. Here, we used AICc to evaluate the relative model fit between a linear, quadratic, and cubic relationship of PM cost over the course of each trial. The Akaike weight calculated from AICc scores from each model are shown in **Figure 2-3a**. We evaluated the lowest AICc for each trial, and then calculated the proportion of trials best fit by either a linear, quadratic, and cubic relationship for each participant (shown in **Figure 2-3b**). A 1st order polynomial (linear model) fit best for nearly all trials (mean = 93.43%, 95% CI = [92.62%, 94.24%]; Akaike weight = 0.873, 95% CI = [0.865, 0.880]), compared to 2nd order fits (mean = 5.72%, 95% CI = [4.99%, 6.45%]; Akaike weight = 0.111, 95% CI = [0.104, 0.119]), or 3rd order fits (mean = 0.85%, 95% CI = [0.68%, 1.02%]; Akaike weight = 0.016, 95% CI = [0.012, 0.019]).

**
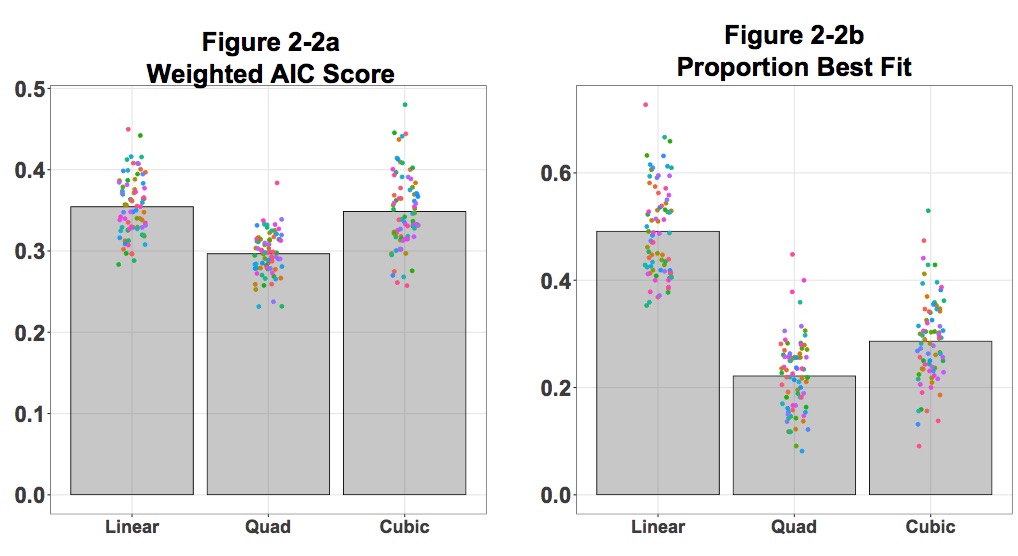
**

**Figure 2-2.** Additional model comparisons for trial-by-trial estimates of PM strategy shifts. One concern with fitting models on a by-trial basis is that noise may bias model selection towards the models with fewer parameters. In order to address this concern, we performed a less conservative AIC (without the small sample correction term) model selection analysis and a bootstrap analysis where polynomial fits were calculated for a random subsample of trials. For the AIC analysis, we performed the same regression analysis steps as detailed for the AICc analysis, but simply used the AIC estimation term instead of the AICc term. After calculating an AIC score for each trial, we then selected the lowest score between the 1^st^, 2^nd^, and 3^rd^ order polynomial as the best for the trial. We then calculated the relative Akaike weight for each model on each trial and average that value for each model type for each participant. Across participants the average Akaike weights were similar between 1^st^ and 3^rd^ order polynomial fits (mean difference = 0.006 (SE = .010), t(77) = 0.65, p=.52; **panel a**). Significantly more trials for each participant were best fit by a 1^st^ order than a 3^rd^ order polynomial (mean difference = 20.5% (SE = 1.7%), t(77) = 12.014, p<.001; **panel b**). Another way to mitigate the impact of noise on model selection is to fit the model on more than a single trial at a time. To avoid averaging across all trials and still getting an estimate of model-fit reliability, we performed a bootstrap analysis. In this analysis, we first z-scored PM-cost values within each subject. Next, we combined all trials into one super-subject. On each bootstrap iteration, 50 trials of each of the five trial types (increasing starting easy, increasing starting middle, fixed, decreasing starting hard, decreasing starting easy) were randomly selected from the super-subject pool. Then, 1^st^, 2^nd^, and 3^rd^ order polynomial models were fit to each trial type sample and the lowest AIC value was selected as the best-fit model type. We repeated this process 1000 times and found that a linear (1^st^ order) polynomial fit a significantly greater number of these samples (57.7% of all trials), followed by a quadratic fit (2^nd^ order, 29.1% of all trials), followed by a cubic fit (3^rd^ order, 13.4% of all trials). This is a significantly greater number of trials selected to be best fit by a linear relationship than would be predicted by chance ($\chi$^2^ (1, n=1000) = 364.06, p <.001).

| **A**  **Ongoing Task Accuracy** |  | **Behavioral Sample (N=50)** | **Neural Sample**  **(N=28)** | **Combined Sample**  **(N=78)** |
| --- | --- | --- | --- | --- |
| Overall | Mean  95% CI | 81.26%  [69.52%,93.00%] | 83.02%  [75.08%, 90.97%] | 83.64%  [75.49%,91.79%] |
|  | | |  |  |
| Difficulty  (1 to 15) | β  95% CI  p | -0.024  [-0.025, -0.023]  <.001** | -0.030  [-0.031, -0.028]  <.001** | -0.026  [-0.027, -0.025]  <.001** |
|  |  |  |  |  |
| Trial Type  (PM, non-PM) | β  95% CI  p | 0.004  [-0.005, 0.012]  0.63 | 0.017  [0.003, 0.030]  0.013* | 0.008  [0.001, 0.016]  0.024* |
|  |  |  |  |  |
| Difficulty x Trial Type | β  95% CI  p | -9.4*10^-5^  [-0.002, 0.002]  0.925 | 0.002  [-0.001, 0.005]  0.224 | 6.2*10^-4^  [-0.001, 0.002]  0.468 |
|  |  |  |  |  |
| Main Effect Model | marginal R^2^ | 0.552 | 0.604 | 0.569 |
|  |  |  |  |  |
| **B**  **Ongoing Task RT** |  |  |  |  |
| Overall | Mean  95% CI | 1.089  [0.895, 1.284] | 1.113  [0.857, 1.369] | 1.098  [0.880, 1.316] |
|  |  |  |  |  |
| Difficulty  (1 to 15) | β  95% CI  p | 0.034  [0.031, 0.036]  < .001** | 0.033  [0.030, 0.037]  < .001** | 0.033  [0.031, 0.036]  < .001** |
|  |  |  |  |  |
| Trial Type  (PM, non-PM) | β  95% CI  p | -0.102  [-0.111, -0.092]  < .001** | -0.139  [-0.153, -0.125]  < .001** | -0.115  [-0.123, -0.107]  < .001** |
|  |  |  |  |  |
| Difficulty x Trial Type | β  95% CI  p | 0.010  [0.008, 0.012]  < .001** | 0.014  [0.011, 0.017]  < .001** | 0.011  [0.010, 0.013]  < .001** |
| Interaction Model | marginal R^2^ | 0.553 | 0.486 | 0.525 |
|  |  |  |  |  |
| **C**  **PM Cost** |  |  |  |  |
| Difficulty  (1 to 15) | β  95% CI  p | -8.217  [-10.471, -5.964]  < .001** | -14.167  [-17.352, -10.982]  < .001** | -10.353  [-12.289, -8.417]  < .001** |
|  |  |  |  |  |
| **D**  **PM Cost Slope** |  |  |  |  |
| Trial Type  (Dec, Fix, Inc) | F  p | F(2,98) = 22.39  < .001** | F(2,54) = 28.53  < .001** | F(2,154) = 47.02  < .001** |
|  |  |  |  |  |
| Decreasing | Mean  95% CI  p(mean>0) | 4.78  [3.41, 6.14]  < .001** | 8.28  [6.25, 10.31]  < .001** | 6.03  [4.86, 7.21]  < .001** |
|  |  |  |  |  |
| Fixed | Mean  95% CI  p(mean>0) | 3.23  [1.59,4.87]  < .001** | 3.53  [1.43, 5.64]  0.002* | 3.34  [2.07, 4.61]  < .001** |
|  |  |  |  |  |
| Increasing | Mean  95% CI  p(mean<0) | -1.03  [-2.34, 0.28]  0.120 | -1.33  [-3.42,0.76]  0.202 | -1.14  [-2.24, -0.42]  0.042* |
|  |  |  |  |  |
| Increasing vs. Fixed | ANOVA  p | F(1,49) = 20.16  < .001** | F(1,27) = 17.38  < .001** | F(1,77) = 37.14  < .001 ** |
|  |  |  |  |  |
| Decreasing vs. Fixed | ANOVA  p | F(1,49) = 3.32  0.075 | F(1,27) = 18.13  < .001** | F(1,77) = 15.10  < .001** |
|  |  |  |  |  |
| Increasing vs. Decreasing | ANOVA  p | F(1,49) = 41.99  < .001** | F(1,27) = 41.02  < .001** | F(1,77) = 78.63  < .001** |
|  |  |  |  |  |
| **E**  **PM Accuracy** |  |  |  |  |
| Overall | Accuracy  95% CI | 69.66%  [50.35%,88.97%] | 83.33%  [73.48%, 93.17%] | 74.57%  [56.82%, 92.31%] |
|  |  |  |  |  |
| Trial Type | ANOVA  p | F(2,98) = 1.19  0.310 | F(2,54) = 0.36  0.699 | F(2,154) = 0.68  0.508 |
|  |  |  |  |  |
| Decreasing | Accuracy  95% CI  p(mean=0) | 70.86%  [64.78%, 76.94%]  < .001** | 82.32%  [77.92%, 86.71%]  < .001** | 74.97%  [70.65%, 79.29%]  < .001** |
|  |  |  |  |  |
| Fixed | Accuracy  95% CI  p(mean=0) | 67.91%  [61.67%, 74.14%]  < .001** | 83.00%  [76.49%, 89.49%]  < .001** | 73.32%  [68.50%, 78.14%]  < .001** |
|  |  |  |  |  |
| Increasing | Accuracy  95% CI  p(mean=0) | 69.39%  [63.99%, 74.80%]  < .001** | 84.61%  [80.28%, 88.95%  < .001**] | 74.86%  [70.77%, 78.94%]  < .001** |
|  |  |  |  |  |
| False Alarm Rate | % of probes | 0. 71%  (SE = 0.11%) | 0.41%  (SE = 0.05%) | 0.60%  (SE = .01%) |
|  |  |  |  |  |
| **F**  **PM Accuracy: Trial Direction * PM Cost Slope** |  |  |  |  |
| Decreasing | β  95% CI =  p | 0.013  [0.003, 0.025]  0.007* | 0.026  [0.008, 0.047]  0.003* | 0.018  [0.008, 0.028]  < .001** |
|  |  |  |  |  |
| Fixed | β  95% CI =  p | 0.018  [0.003, 0.034]  0.011* | 0.025  [-0.003, 0.058]  0.041* | 0.020  [0.006, 0.033]  0.003* |
|  |  |  |  |  |
| Increasing | β  95% CI =  p | -0.006  [-0.016, 0.004]  0.126 | -0.003  [-0.025, 0.018]  0.401 | -0.006  [-0.015, 0.004]  0.119 |
|  |  |  |  |  |
| Decreasing vs. Increasing | β  95% CI =  p | 0.019  [0.005, 0.034]  0.006* | 0.0290  [-0.002, 0.061]  0.031* | 0.024  [0.011, 0.037]  < .001 ** |
|  |  |  |  |  |
| Decreasing vs. Fixed | β  95% CI =  p | 0.005  [-0.013, 0.022]  0.305 | -0.001  [-0.035, 0.035]  0.534 | 0.001  [-0.015, 0.017]  0.423 |
|  |  |  |  |  |
| Increasing vs. Fixed | β  95% CI =  p | 0.024  [0.005, 0.0421]  0.007* | 0.028  [-0.003, 0.061]  0.040* | 0.025  [0.009, 0.041]  0.025* |
|  |  |  |  |  |
| * p < 0.05, ** p < 0.001 |  |  |  |  |

**Table 2-3. Comparison of behavioral results from experiments 1 and 2.** These data include the key results presented in **Figure 2** separately for the behavioral-only participants, the neural participants, and the combined groups. Each analysis section of the table (A-F) corresponds to the same panel from **Figure 2**. Analyses of the relationship between trial type (PM/non-PM), Difficulty (1 to 15) and OG task accuracy, OG RT, and PM cost were carried out by first running a mixed-effect regression using the *lme4* package in R of the interaction between trial type and difficulty and then a separate model comparing the main effects of difficulty and trial type without the interaction term. Random effects of individual slope and intercept were included in each regression. Within-subject ANOVAs were used to compare PM cost slope and PM accuracy across conditions.

| **Cluster Index** | **Z** | **x** | **y** | **z** | **ROI Label** | **Hemisphere** |
| --- | --- | --- | --- | --- | --- | --- |
| 5 | 8.07 | 10 | -98 | 6 | Occipital Pole | R |
| 5 | 7.83 | -26 | -58 | -8 | Fusiform | L |
| 5 | 7.5 | 32 | -56 | -14 | Fusiform | R |
| 5 | 7.36 | -4 | -72 | -24 | Cerebellum | L |
| 5 | 7.24 | -18 | -62 | 8 | Intracalcarine Cortex | L |
| 5 | 7.2 | 6 | -70 | -26 | Cerebellum | R |
| 4 | 4.98 | 36 | 36 | 32 | dlPFC (BA 9) | R |
| 4 | 4.95 | 34 | 54 | 14 | rlPFC (BA10) | R |
| 4 | 4.84 | 24 | 56 | 8 | rlPFC (BA10) | R |
| 4 | 4.75 | 36 | 42 | 34 | dlPFC (BA 9) | R |
| 4 | 4.75 | 38 | 52 | 14 | rlPFC (BA10) | R |
| 4 | 4.74 | 28 | 60 | 8 | rlPFC (BA10) | R |
| 3 | 5.85 | 34 | 22 | 10 | anterior insula | R |
| 3 | 5.26 | 32 | 28 | -2 | anterior insula | R |
| 3 | 5.24 | 32 | 26 | 2 | anterior insula | R |
| 3 | 5.19 | 36 | 28 | 10 | anterior insula | R |
| 3 | 5.18 | 42 | 18 | 6 | anterior insula | R |
| 3 | 4.99 | 38 | 16 | 24 | anterior insula | R |
| 2 | 5.51 | -8 | -38 | 24 | posterior cingulate | L |
| 2 | 5.44 | 8 | -22 | 26 | posterior cingulate | R |
| 2 | 5.31 | -8 | -24 | 26 | posterior cingulate | L |
| 2 | 5.25 | -6 | -28 | 26 | posterior cingulate | L |
| 2 | 5.17 | -8 | -44 | 20 | posterior cingulate | L |
| 2 | 5.13 | -6 | -18 | 28 | posterior cingulate | L |
| 1 | 4.75 | -30 | 50 | 10 | rlPFC (BA10) | L |
| 1 | 4.71 | -26 | 42 | 6 | rlPFC (BA10) | L |
| 1 | 4.43 | -34 | 58 | 14 | rlPFC (BA10) | L |
| 1 | 4.35 | -36 | 54 | 20 | rlPFC (BA10) | L |
| 1 | 4.05 | -34 | 58 | 20 | rlPFC (BA10) | L |
| 1 | 3.59 | -22 | 50 | -4 | rlPFC (BA10) | L |

**Table 3-1: PM > Non-PM GLM Analysis.** FSL’s FEAT was used to identify voxels that were more responsive on PM trials than on non-PM trials (cluster correction, p < .001). This table lists the coordinates and descriptors for all significant voxel clusters.


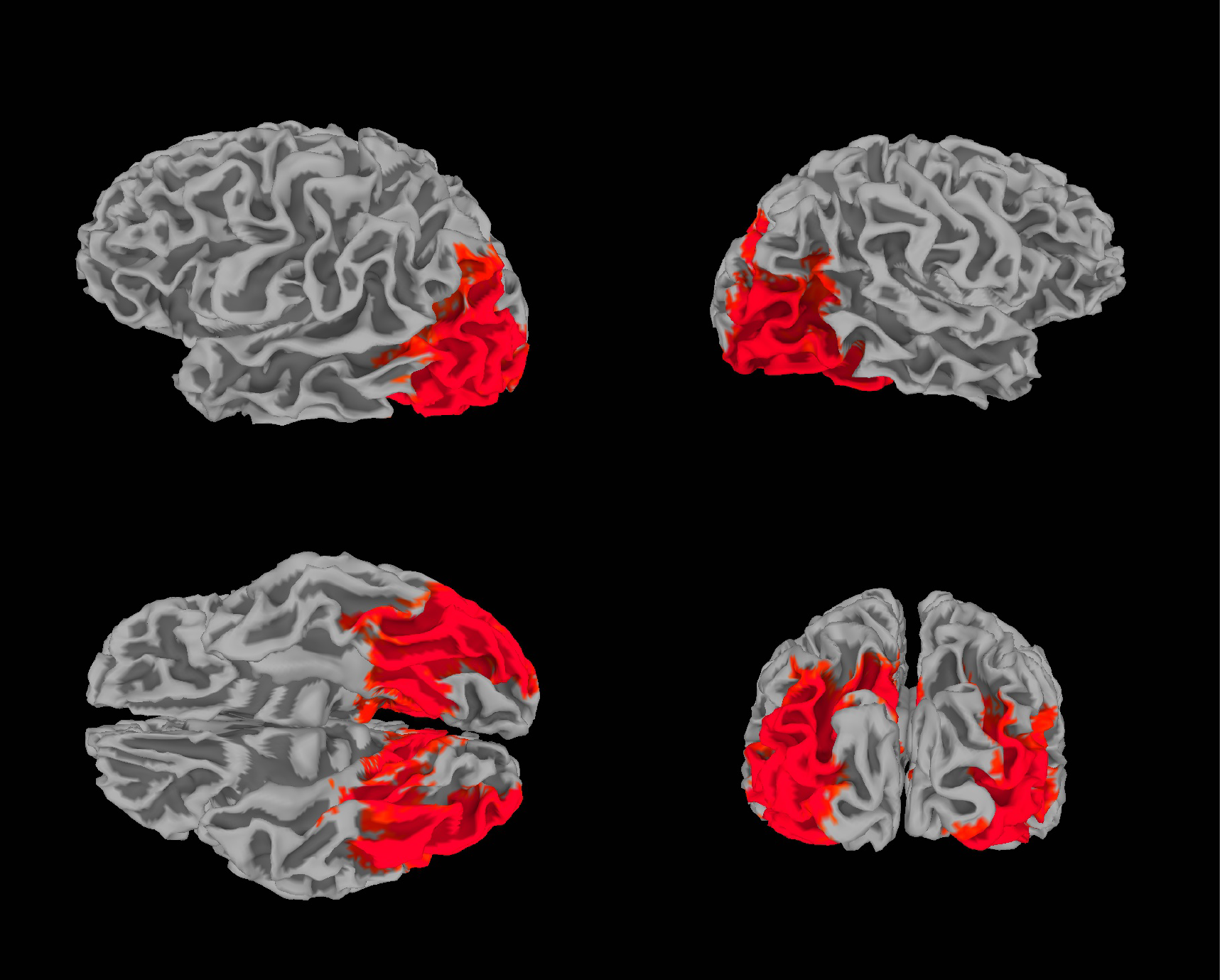


**Figure 3-2.** **Surface searchlight analysis.** Results from the surface-based searchlight classification analysis to decode the PM intention on PM trials. Vertices in red indicate those that survived threshold-free cluster enhancement significance testing (H_0_ mean = 50%, p<.001). Results indicate that classification was successful only in two particular posterior regions: the ventral temporal cortex and lateral occipital cortex. Notably absent from this map is the anterior lateral prefrontal cortex. To perform this analysis, anatomical surface outputs from Freesurfer *recon-all* were converted to AFNI/SUMA format using *SUMA_Make_Spec_FS*. Surfaces were remapped to a standard topology using *MapIcosahedron* and co-registered to a reference functional volume using *align_epi_anat* so that functional data could be masked by the surface volume. Voxels determined to not be part of the surface were masked out of the searchlight analysis. Surface searchlight analysis was performed in MATLAB using functions from the CosMoMVPA toolbox. Each searchlight sphere was determined by selecting the 100 closest vertices to a center vertex according to geodesic distance. L2-weighted logistic regression classifiers were trained on four categories and tested within each searchlight sphere using a k-fold cross validation procedure. Only the five main PM task blocks were used for this analysis, and data from the localizers were excluded. That meant that on each k-fold iteration, 4 out of 5 PM-task blocks were used for training the classifier, and one held out block was used for testing. Accuracy was then computed on face and scene probes. The face/scene accuracy across all five folds was averaged and that value was assigned to the center vertex of that sphere.
